# Supplementary material for: Determining the impact of an artificial intelligence tool on the management of pulmonary nodules detected incidentally on CT (DOLCE) study protocol: a prospective, non-interventional multicentre UK study
Source: BMJ Open. 2024 Jan 4;14(1):e077747. doi: 10.1136/bmjopen-2023-077747 (PMC10773382; doi:10.1136/bmjopen-2023-077747)
Supplement: Author's manuscript [file bmjopen-2023-077747.draft_revisions.pdf]

BMJ Open

BMJ Open is committed to open peer review. As part of this commitment we make the peer review history of every article we publish publicly available.

When an article is published we post the peer reviewers' comments and the authors' responses online. We also post the versions of the paper that were used during peer review. These are the versions that the peer review comments apply to.

The versions of the paper that follow are the versions that were submitted during the peer review process. They are not the versions of record or the final published versions. They should not be cited or distributed as the published version of this manuscript.

BMJ Open is an open access journal and the full, final, typeset and author-corrected version of record of the manuscript is available on our site with no access controls, subscription charges or pay-per-view fees (http://bmjopen.bmj.com).

If you have any questions on BMJ Open's open peer review process please email info.bmjopen@bmj.com

BMJ Open

Determining the impact of an artificial intelligence tool on the management of pulmonary nodules detected incidentally on computed tomography: DOLCE study protocol

Journal:	*BMJ Open*
Manuscript ID	bmjopen-2023-077747
Article Type:	Protocol
Date Submitted by the Author:	14-Jul-2023
Complete List of Authors:	O'Dowd, Emma; Nottingham University Hospitals NHS Trust<br>Berovic, Marko; King's College Hospital NHS Foundation Trust<br>CALLISTER, Matthew; Leeds Teaching Hospitals NHS Trust<br>Chalitsios, Christos ; University of Nottingham<br>Chopra, Disha; Optellum Ltd<br>Das, Indrajeet; University Hospitals of Leicester NHS Trust<br>Draper, Adrian; St George's Hospital, Respiratory Medicine<br>Garner, Justin; Royal Brompton and Harefield Hospitals<br>Gleeson, Fergus; Oxford University Hospitals NHS Foundation Trust<br>Janes, Sam; University College London<br>Kennedy, Martyn; Leeds Teaching Hospitals NHS Trust,<br>Lee, Richard; Royal Marsden Hospital NHS Trust<br>Mauri, Fabrizio; Optellum Ltd<br>McKeever, Tricia; University of Nottingham<br>McNulty, William; King's College Hospital NHS Foundation Trust<br>Murray, James; Royal Free London NHS Foundation Trust<br>Nair, Arjun; University College Hospital<br>Park, John; Oxford University Hospitals NHS Foundation Trust<br>Rawlinson, Janette; National Cancer Research Institute, Consumer forum, NCRI CSG (lung) subgroup, BTOG Steering Committee, NHSE CEG;<br>Sago, Gurdeep; University of Newcastle upon Tyne<br>Scarsbrook, Andrew; Leeds Teaching Hospitals NHS Trust<br>Shah, Pallav; Royal Brompton and Harefield NHS Foundation Trust<br>Sudhir, Rajini; University Hospitals of Leicester NHS Trust<br>Talwar, Ambika; Oxford University Hospitals NHS Foundation Trust<br>Thakrar, Ricky; University College London Hospitals NHS Foundation Trust<br>Watkins, Johnathan; Optellum Ltd<br>Baldwin, David; Nottingham University Hospitals NHS Trust
Keywords:	Clinical Trial, Chest imaging < RADIOLOGY & IMAGING, RADIOLOGY & IMAGING, Computed tomography < RADIOLOGY & IMAGING, Respiratory tract tumours < THORACIC MEDICINE

For peer review only - http://bmjopen.bmj.com/site/about/guidelines.xhtml

Title

Determining the impact of an artificial intelligence tool on the management of pulmonary nodules detected incidentally on computed tomography: DOLCE study protocol

Emma L O'Dowd[1], Marko Berovic[2], Matthew Callister[3], Christos V. Chalitsios[4], Disha Chopra[5], Indrajeet Das[6], Adrian Draper[7], Justin L Garner[8], Fergus Gleeson[9], Sam Janes[10], Martyn Kennedy[3], Richard Lee[11], Fabrizio Mauri[5], Tricia McKeever[4], William McNulty[2], James Murray[12], Arjun Nair[13], John Park[9], Janette Rawlinson[14], Gurdeep S Sagoo[15], Andrew Scarsbrook[3], Pallav L Shah[8], Rajini Sudhir[6], Ambika Talwar[9], Ricky Thakrar[13], Johnathan Watkins[5], David R Baldwin[1]

1. Nottingham University Hospitals NHS Trust, Nottingham, NG5 1PB

2. King's College Hospital NHS Foundation Trust, London SE5 9RS

3. Leeds Teaching Hospitals NHS Trust, Leeds LS9 7TF

4. University of Nottingham, Nottingham, NG5 1PB

5. Optellum Ltd, Oxford Centre For Innovation, New Road, Oxford, England, OX1 1BY

6. University Hospitals of Leicester NHS Trust, Leicester, Leicester, LE3 9QP

7. St George's University Hospitals NHS Foundation Trust, London, SW17 0QT

8. Royal Brompton and Harefield hospitals, London SW3 6NP

9. Oxford University Hospitals NHS Trust, Oxford, UK

10. Lungs for Living Research Centre, University College London, London, UK

11. The Royal Marsden NHS Foundation Trust, London SW3 6JJ

12. Royal Free London NHS Foundation Trust, London NW3 2QG

13. University College London Hospitals NHS Foundation Trust, London

14. Patient representative, National Cancer Research Institute (NCRI), Consumer forum, NCRI CSG (Lung) Subgroup, BTOG Steering Committee, NHSE CEG, UK

15. University of Newcastle, Newcastle upon Tyne

Corresponding author: Emma L O'Dowd (emma.odowd@nottingham.ac.uk)

For peer review only - http://bmjopen.bmj.com/site/about/guidelines.xhtml

Abstract

Introduction

In a small percentage of patients, pulmonary nodules found on computed tomography (CT) scans are early lung cancers. Lung cancer detected at an early stage has a much better prognosis. The British Thoracic Society (BTS) guideline on managing pulmonary nodules recommends using multivariable malignancy risk prediction models to assist in management. Whilst these guidelines seem to be effective in clinical practice, recent data suggest that artificial intelligence (AI)-based malignant-nodule prediction solutions might outperform existing models.

Methods and analysis

This study is a prospective, observational multicentre study to assess the clinical utility of an AI-assisted CT-based lung cancer prediction tool (LCP) for managing pulmonary nodule patients versus standard care. Two thousand patients will be recruited from 10 different UK hospitals. The primary outcome is the difference between standard care and LCP-guided care in terms of the rate of benign-nodules and cancer patients discharged straight after the assessment of the baseline CT scan. Secondary outcomes investigate adherence to clinical guidelines, other measures of changes to clinical management, patient outcomes, and cost-effectiveness.

Ethics and dissemination

This study has been reviewed and given a favourable opinion by the South Central - Oxford C Research Ethics Committee in UK (REC reference number: 22/SC/0142).

Study results will be available publicly following publication in open-access journals. Researchers will also contact participants directly to provide a link to the study results.

Trial registration number

The study is registered in Clinicaltrials.gov, number: NCT05389774

Strengths and limitations

- This is the first prospective study examining the real-world deployment and use of artificial intelligence (AI)-based computer-aided diagnosis (CADx) for pulmonary nodules
- The study endpoints go beyond diagnostic accuracy and examine whether the AI-based CADx tool could help change the clinical management of patients and help improve patient comes and the cost-effectiveness of pulmonary nodule management
- Data collected will also be used to examine whether the AI-based CADx tool could be used as a binary recommendation-based tool
- The study is observational in nature
- The analysis of some of the secondary objectives is exploratory due to potential sample size restrictions

Keywords

Clinical trial; chest imaging; radiology & imaging; computed tomography; respiratory tract tumours

For peer review only

Introduction

From 2017 to 2019, lung cancer caused 34,771 deaths in the UK per annum, more than prostate and bowel cancer combined (28,846), the next most common causes of cancer death. Over the same period, there were 24% more deaths in women from lung cancer than from breast malignancy (14,140 vs 11,415) [1]. This is because almost three-quarters of people with lung cancer present with stage III or IV disease when the prognosis is poor despite recent advances in systemic anti-cancer treatment. Lung cancer detected at an early stage, usually by thoracic computed tomography (CT), has a much better prognosis. Screening with low-dose CT thorax has recently been confirmed to reduce lung cancer and all-cause mortality [2–4]. Central to the success of screening is the accurate management of pulmonary nodules, which may represent early-stage lung cancer, but are far more often benign. Pulmonary nodules are also a common finding on CT scans done for other reasons. In some settings, this may yield more early lung cancers than screening. Identifying malignant pulmonary nodules on CT scans is the primary way to detect lung cancer at stage I, when the prognosis is best. By definition, isolated pulmonary nodules without other lung changes, are stage I if proven to be lung cancer. Currently, most nodules are detected outside screening programmes in the UK, providing an important rationale for this study to address the effective management of these incidentally detected nodules.

The British Thoracic Society (BTS) guideline on managing pulmonary nodules was published in 2015. This guidance was the first to recommend semi-automated volumetry as the standard for nodule measurement and the use of multivariable malignancy risk prediction models to assist in management. Whilst these guidelines seem to be effective in clinical practice [5], recent data suggest that artificial intelligence (AI)-based malignant-nodule prediction solutions appear to outperform existing multivariable models [6,7]. This could result in a considerable reduction in the number of follow-up CT, which is much needed in the NHS, where current and forecasted demand for radiology services exceeds capacity [8,9].

Optellum Ltd has developed and extensively validated an AI-based computer-aided diagnostic (CADx) tool for lung cancer prediction (LCP) in pulmonary nodules, which has UK Conformity Assessed marking under UK medical device regulations. The AI model underpinning the LCP is a convolutional neural network (CNN), referred to hereafter as the 'LCP-CNN'. LCP-CNN was derived and internally validated using data from diverse populations from the US and UK. The model was then externally validated in two independent cohorts of patients with incidentally detected pulmonary nodules, amounting to over 1,500 patients in aggregate [6,7]. The LCP-CNN demonstrated greater accuracy in these studies than the Brock and Mayo malignancy risk models (used in managing incidentally detected nodules in the UK and US, respectively). The product is provided within the "Virtual Nodule Clinic" (VNC).

The study aims to assess this new AI tool in a prospective real-world setting in the UK. This will be done by calculating malignancy risk using LCP and measuring how management would differ if it were governed by the risk provided. The aim is to provide evidence as to whether LCP should be the new standard of care in pulmonary nodule management.

Methods and analysis

The study acronym is "DOLCE" (Determining the impact of Optellum's LCP artificial intelligence solution on service utilisation, health Economics and patient outcomes).

For peer review only - http://bmjopen.bmj.com/site/about/guidelines.xhtml

Design and setting

DOLCE is a multi-centre prospective observational cohort study recruiting patients with 5-30 mm solid and part-solid pulmonary nodules incidentally detected on thoracic CT scans performed as part of routine practice. The study aims to recruit 2,000 patients from multiple centres.

Patients will be asked to consent to and be enrolled at ten acute NHS hospital trusts in England. A recruitment period of one year is planned, with one year of follow-up to collect data and ascertain the ground-truth diagnosis of recruited patients.

Clinical pathway

This is a non-interventional study. There will be no anticipated change to the standard of care the recruited patients receive throughout the study's duration. The potential impact of AI-modified management will be assessed by comparing routine clinical management with that which would have been conditional upon the LCP risk score. The scores will be provided to clinicians after routine management decisions have been made, either during the same MDT meeting or at a separate research meeting. A hypothetical clinical decision based on the LCP score will then be recorded.

Study population

Patients who have undergone CT that has detected a pulmonary nodule will be reviewed for study eligibility (Table 1).

Table 1: Patient eligibility criteria

**Inclusion criteria**
Patients aged 35 years or over
Patients with a baseline CT study with at least one incidentally detected solid or part-solid pulmonary nodule that is 5-30mm in maximum axial diameter for the whole lesion, measured using manual electronic callipers, and that is not fully calcified. The solid component of the part-solid nodule must be ≥ 80% of the total nodule size.
Patients with a baseline CT study which includes at least one series with acquisition parameters which meet VNC requirements
The patient has a baseline CT study that includes at least one series that comprises at least one full-inspiration breath-hold scan without a high degree of contrast media and does not exhibit quality issues (e.g., motion artefacts)
**Exclusion criteria**
Patients who received a diagnosis of cancer in the last 5 years
Patients who have thoracic implants that adversely impact the imaging appearances of the nodule
Patients who have more than five reported pulmonary nodules of any size or type, excluding fully calcified nodules (a marker of prior granulomatous infection or metastatic lung cancer)
Patients who have one or more additional nodules that are already undergoing follow-up according to pulmonary nodule management standard care

For peer review only - http://bmjopen.bmj.com/site/about/guidelines.xhtml

Patients who have one or more additional nodules that are pure ground glass opacity (GGO) of 5mm in maximum axial diameter for the whole lesion measured using manual electronic callipers
The patient has one or more additional nodules >30mm in maximum axial diameter for the whole lesion measured using manual electronic callipers.
Patients with part-solid nodules where the solid component is < 80% of the total nodule size.

AI model

The output of the LCP is a risk score that is an integer from 1 to 10 and forms part of Optellum's VNC patient management software. A score of 1 indicates the nodule is highly likely to be benign, while a score of 10 is most likely to be malignant. The AI model underpinning the LCP is the LCP-CNN, the raw score available to researchers as a continuous value from 0 to 100, as previously reported [6,7].

In this study, the LCP will be compared to standard care in three ways:

- LCP as an integer (1-10) risk score to aid physician decision-making (physician & AI)
- LCP-CNN alone as a threshold-based rule-out recommendation (AI alone)
- LCP-CNN combined with a threshold-based rule-out recommendation to aid physician decision-making (physician & AI)

Data collection

Study teams at the ten NHS Trusts from which patients will be recruited will identify eligible patients and obtain informed consent. Consent will be obtained verbally (for patients who would not normally come on-site as part of standard care) or via written consent. Consent will be sought to analyse standard-care CT scans, associated clinical meta-data collection, and the health economics assessment.

Once a patient has given consent and enrolled into the study, their baseline CT scan will be sent to VNC. Authorised research staff will then log into VNC, select the nodules of interest, and record the LCP score for that nodule. The local standard nodule service decision-makers will provide a standard-care decision for eligible, consented patients via their usual nodule management pathway. The standard-care decision for eligible consented patients will then be recorded in an electronic Case Report Form (eCRF) without knowing the LCP score. The clinical team will thereafter be given the LCP score and relevant patient data in the nodule MDT or as part of a separate research meeting. Their decision on the next management step, had they been guided by the LCP score, will then be recorded in the eCRF. The clinical team will have the option to modify management at this point. Any modification of management will be recorded in the eCRF.

Subsequent follow-up images for each patient will be sent to the VNC over the course of their nodule follow-up, and the final nodule diagnosis will be recorded. Since standard patient care can sometimes deviate from BTS guidelines, all participating sites will also collect information on patient and nodule characteristics necessary to follow the BTS guidelines. In this way, AI-guided management can be compared with BTS-compliant management. Other data points

For peer review only - http://bmjopen.bmj.com/site/about/guidelines.xhtml

necessary to evaluate service utilisation (e.g. additional scans, biopsies, and treatments for lung nodules or lung cancers) will also be recorded in the eCRF.

Evaluation of the threshold-based tests, which require access to LCP-CNN values, will be performed as a batch at the end of the study follow-up period. The threshold will be defined prospectively based on values from training datasets of similar patient populations. The use of the recommendation with physicians making the decision will be evaluated via a separate research meeting at the end of the 12-month follow-up period.

For cost-effectiveness modelling, quality of life data will be collected by inviting patients to complete an EQ-5D-5L quality of life questionnaire at two-time points: a) as close as possible to the baseline CT scan on which their pulmonary nodule was detected; and b) at a 1-year timepoint afterwards. A participant diary about primary care usage will also be collected at the one-year mark.

Ground truth diagnosis

Patients will be classified into whether they have benign or malignant nodules as follows:

1. Benign-nodule patients:
    a. By resolution or stability of all qualifying incidental pulmonary nodules as determined from volumetry-based measurement on imaging follow-up over 12 months
    b. By histopathology from biopsy or surgery of all qualifying incidental pulmonary nodules (within 12 months of baseline CT scan)
2. Malignant-nodule patients:
    a. By histopathology from biopsy or surgery (within 12 months of baseline CT scan) of at least one qualifying incidental pulmonary nodule
3. Probable benign-nodule patients:
    a. By resolution, stability, or shrinkage of qualifying incidental pulmonary nodules that are not in ground-truth outcomes #1 or #2 above as determined from volumetry-based measurement on imaging follow-up conducted between 3-12 months after the baseline CT scan
    b. By resolution, stability, or shrinkage of qualifying incidental pulmonary nodules that are not in ground-truth outcomes 1 or 2 above as determined from diameter-based measurement on imaging follow-up conducted 12 months after the baseline CT scan (supplemented with a volume-based measurement of the nodule that will be performed for research purposes)
4. Probable malignant-nodule patients:
    a. By clinical diagnosis of lung cancer, in whom a biopsy or resection is not possible, based on high clinical probability and who are registered at a lung cancer MDT/and/or receive non-surgical treatment (SABR/ radiotherapy/ microwave ablation)

Volumetry-based measurement of nodule stability – and hence a benign diagnosis – will be satisfied if the lung nodule volume on the follow-up CT scan was <125% of the lung nodule volume on the baseline CT scan.

For peer review only - http://bmjopen.bmj.com/site/about/guidelines.xhtml

If the standard care at a particular site does not incorporate volumetry-based measurements, the volumetric measurement will be applied as part of the research study to the relevant follow-up CT scans.

Any lung nodule patients who do not fall into either of the two ground-truth outcomes 1 or 2 above will be considered to have an indeterminate diagnosis at the time of analysis and will be analysed with and without the inclusion of the ground-truth outcomes in 3 and 4.

Outcome measures

The outcome measures for the primary and secondary objectives are outlined in Table 2.

Table 2: Study objectives and outcome measures

Objective	Outcome measure
**Primary**:<br>Determine the potential effect of the LCP on discharge	The measured difference between standard care, LCP, and LCP-guided care for:<br>• Number and percentage of cancer patients discharged (straight after assessment of the baseline scan)<br>• Number and percentage of benign-nodule patients discharged (straight after assessment of the baseline scan)
**Secondary**:<br>Determine the potential effect of the LCP on possible adherence to clinical guidelines	The measured difference between standard care and LCP-guided care for:<br>• Number and percentage of patients for whom a validated risk model (Brock or LCP) is used to guide the next clinical management step (i.e., counting the instances where Brock is not used or where LCP is not possible to compute, or it is ignored)
**Secondary**:<br>Determine the potential effect of the LCP on overall clinical management, as well as scan and procedure utilisation	Number and percentage of cancer patients for whom there would have been a change in clinical management by LCP and LCP-guided care compared with the actual (standard) care (correctly for more aggressive management and incorrectly for less aggressive management).<br>Number and percentage of benign-nodule patients for whom there would have been a change in clinical management by LCP and LCP-guided care compared with the actual (standard) care (incorrectly by more aggressive management and correctly for less aggressive management).<br>The measured difference between standard care, LCP, and LCP-guided care for:<br>• Number and percentage of CT scans and PET/CT scans performed on benign-nodule patients<br>• Number and percentage of non-surgical biopsies performed on benign-nodule patients<br>• Number and percentage of surgical excisions on benign-nodule patients

For peer review only - http://bmjopen.bmj.com/site/about/guidelines.xhtml

**Secondary**: Determine the potential effect of LCP-informed care versus standard care on patient outcomes	The measured difference between standard care, LCP, and LCP-guided care for: • Number and percentage of thoracic, respiratory, or vascular events related to biopsies or surgical excisions for lung nodules or suspected lung cancer occurring within 30 days of the procedure on benign-nodule patients • Number and percentage of lung cancers stratified by stage • Time in days between nodule detection and lung cancer diagnosis
**Secondary**: Determine the potential health-economic effect of LCP-informed care versus standard care	The measured difference between standard care, LCP, and LCP-guided care for: • The composite standardised GBP costs of all healthcare-related activity for lung nodules or suspected lung cancer • Health-Related Quality of Life (using EQ5D5L to generate QALYs) • Estimate cost-effectiveness (Incremental costs per QALY gained)

Statistical analysis

Sample size

For calculations relating to the primary objectives of a measured difference between standard care and LCP for*:*

- Number and percentage of cancer patients discharged: results from thresholds defined for discharge in a previous study are used [6]. Based on previous data within the cancer group, assuming for the discordant pairs that the Brock model threshold (representing the standard of care) identified 0.4% of cancers for which the LCP threshold would have recommended discharge, and the LCP threshold correctly identified 2.6% of cancers for which the Brock model threshold would have recommended discharge, then using McNemar's test with 90% power and a significance level of 0.05, 659 participants would be needed. However, with a sample size of 2000, there is 90% power to detect a difference between the methods if the percentages were as small as 1.0% and 2.3%, respectively
- Number and percentage of benign-nodule patients discharged: again, results from thresholds defined for discharge in a previous study are used [6]. Based on previous data within the benign group, assuming for the discordant pairs that 17.0% of benign nodules are recommended for discharge by the LCP threshold but not by the Brock model threshold. In comparison, 13.5% are recommended for discharge by the Brock model threshold but not by the LCP threshold; there is 80% power to detect a significant difference between the two tests with 2,026 individuals using McNemar's test. This equates to an odds ratio of 1.26.

Analysis

Statisticians will carry out all analyses. For each outcome measure, the following will be reported:

For peer review only - http://bmjopen.bmj.com/site/about/guidelines.xhtml

- The number of participants included in the analysis, stratified by the relevant group
- Mean or median (SD or IQR) for continuous outcomes, and numbers and proportions (percentage) for binary outcomes
- An effect size (odds ratio) with a 95% confidence interval
- A two-sided p-value

For both primary objectives, measured differences between standard care and LCP for cancer patients diagnosed and benign-nodule patients discharged will be analysed using conditional logistic regression. Secondary outcomes will be described and analysed according to their distribution.

Health economics analysis

The economic analysis will estimate the expected costs and expected QALYs gained from standard care, LCP, and LCP-guided care over patient lifetime from the perspective of the UK NHS. Incremental analyses will identify the most cost-effective permutation with exploratory analyses undertaken to determine the uncertainty around measures of cost-effectiveness. Costs will be calculated as quantities of resource use multiple by appropriate unit costs (e.g. NHS Reference Costs) and will be specific to a year to be determined at time of analysis. A HEAP (health economics analysis plan) will be produced prior to undertaking the analysis at the end of the study patient follow-up period.

Patient and public involvement

An extensive programme of patient and public involvement (PPI) input was arranged to provide input into the study objectives, design, materials and governance.

Specifically, a PPI representative, who sits on the Trial Management Group, provided input from study conception to developing study materials. This guidance and support will continue during the management and conduct of the study. An independent PPI contributor is a member of the trial steering committee (TSC). Further input was gathered from a group of PPI contributors selected from across the country with varying experiences of lung nodules, cancer or none from a patient and/or carer perspective during facilitated virtual workshops.

Input received to date has ranged from considerations of healthcare inequalities and other biases, awareness of AI generally and in healthcare, methods of and materials for participant recruitment and communication, and the design of any data collected during the study.

Ethics and dissemination

This study has been reviewed and given a favourable opinion by the South Central - Oxford C Research Ethics Committee in UK (REC reference number: 22/SC/0142).

Given the study's observational nature, we do not envisage any elevation to the harms or the risk of harm for patients. The sponsor and Chief Investigator have conducted a full risk assessment.

Patients will be asked to give their consent to participate in the study (including the analysis of standard-care CT scans, associated data collection and the healthcare economics assessment) by telephone or face-to-face when attending routine clinic appointments. No additional patient visits, scans or procedures are planned for the study. Patients not wishing

For peer review only - http://bmjopen.bmj.com/site/about/guidelines.xhtml

to participate in the health economics assessment may still participate in the study's CT imaging analysis.

Discussion

The DOLCE study will address several important pulmonary nodule risk assessment and management issues. The majority (>95%) of pulmonary nodules are benign [4]. The challenge in nodule management is to identify malignant nodules early whilst minimising harm from over-investigating benign nodules and reducing variability in reporting/ management between individual clinicians. An AI-based CADx model, in the hands of trained respiratory physicians or thoracic radiologists, can help address these challenges. An AI model can account for nodule size, shape, location and other radiological factors consistently and without requiring subjective judgement or data entry on the part of the reading respiratory physician or radiologist [6]. Moreover, the ability to systematically train and refine the model on tens of thousands of images with a known diagnosis confers AI models an advantage in terms of diagnostic accuracy over non-AI models [10]. LCP-CNN has been externally validated in representative populations and has been shown to improve the current BTS guideline approach [6]. It has also been shown that respiratory physicians and radiologists interpreting pulmonary nodules on CT scans with the aid of the LCP-CNN make more accurate predictions and appropriate clinical decisions than without it. In the same study, the LCP-CNN also improved the consistency of malignancy-risk estimation and clinical decision-making between physician readers of a CT scan [11].

However, the previous work does not answer whether the superior performance of the LCP-CNN (compared with standard care) translates into clinical benefit for the NHS and patients with 5-30mm pulmonary nodules. Moreover, no previous studies have fully explored the utility of the AI-based LCP (the underpinning AI model of which is the LCP-CNN) when installed as software at local sites and presented to local nodule specialists in a way that mimics how it might be deployed in real world clinical practice.

This study, will define to what extent the greater consistency and accuracy conferred by the validated AI-underpinning LCP translates into clinical benefit. This will look beyond diagnostic performance and quantify the impact of LCP usage on the proportion of scans and invasive procedures on benign-nodule patients and the accompanying harms and costs to the NHS. This closely replicates how the LCP would be deployed if used in standard care.

References

1 Cancer Research UK. Cancer mortality for common cancers. 2022.https://www.cancerresearchuk.org/health-professional/cancer-statistics/mortality/common-cancers-compared

2 Lindholt JS, Søgaard R. Lung-Cancer Screening and the NELSON Trial. *N Engl J Med* 2020;**382**:2164. doi:10.1056/NEJMc2004224

3 de Koning HJ, van der Aalst CM, de Jong PA, *et al.* Reduced Lung-Cancer Mortality with Volume CT Screening in a Randomized Trial. *N Engl J Med* 2020;**382**:503–13. doi:10.1056/NEJMoa1911793

For peer review only - http://bmjopen.bmj.com/site/about/guidelines.xhtml

4 National Lung Screening Trial Research Team, Aberle DR, Adams AM, *et al.* Reduced lung-cancer mortality with low-dose computed tomographic screening. *N Engl J Med* 2011;**365**:395–409. doi:10.1056/NEJMoa1102873

5 Balata H, Ruparel M, O'Dowd E, *et al.* Analysis of the baseline performance of five UK lung cancer screening programmes. *Lung Cancer* 2021;**161**:136–40. doi:10.1016/j.lungcan.2021.09.012

6 Baldwin DR, Gustafson J, Pickup L, *et al.* External validation of a convolutional neural network artificial intelligence tool to predict malignancy in pulmonary nodules. *Thorax* 2020;**75**:306–12. doi:10.1136/thoraxjnl-2019-214104

7 Massion PP, Antic S, Ather S, *et al.* Assessing the Accuracy of a Deep Learning Method to Risk Stratify Indeterminate Pulmonary Nodules. *Am J Respir Crit Care Med* 2020;**202**:241–9. doi:10.1164/rccm.201903-0505OC

8 The Royal College of Radiologists. RCR Clinical radiology census report 2021. 2021.

9 Maskell G. Why does demand for medical imaging keep rising? *BMJ* 2022;:o2614. doi:10.1136/bmj.o2614

10 LeCun Y, Bengio Y, Hinton G. Deep learning. *Nature* 2015;**521**:436–44. doi:10.1038/nature14539

11 Kim RY, Oke JL, Pickup LC, *et al.* Artificial Intelligence Tool for Assessment of Indeterminate Pulmonary Nodules. *Radiology* 2022.

For peer review only

Authors' contributions

All authors contributed to the development and set up of the study. EOD, DRB, JW, FM wrote the manuscript, all authors contributed to, reviewed and approved the final version of the manuscript.

Funding statement

This work is supported NHS Accelerated Access Collaborative. Grant number AI_AWARD02266. Sponsor organisation is Nottingham University Hospitals NHS Trust. The sponsor organisation and funder have had no role in study design.

Competing interests

FG reports grant funding from NIHR and Innovate UK, consultancy fees from Polarean Ltd and has shares in Optellum Ltd; SJ has received grant funding from GRAIL Ltd, consultancy fees/ honoraria from AstraZeneca and Chiesi, stock options in Optellum Ltd and his spouse is employed by AstraZeneca; RL is funded by the Royal Marsden NIHR BRC, Royal Marsden Cancer charity and SBRI (including QURE.AI). RL's institution receives compensation for time spent in a secondment role for the lung health check program and as a National Specialty Lead for the National Institute of Health and Care Research. He has received research funding from CRUK, Innovate UK (co-funded by GE Healthcare, Roche and Optellum), SBRI, RM Partners Cancer Alliance and NIHR (co-applicant in grants with Optellum). He has received honoraria from CRUK; WM has received honoraria from Amgen; AN declares grants from

For peer review only - http://bmjopen.bmj.com/site/about/guidelines.xhtml

Department of Health's NIHR Biomedical Research Centre's funding scheme and GRAIL, consulting fees from Aidence BV, Faculty Science LTD and MSD; Support for attending meetings from Takeda Limited; Advisory Board participation for Aidence BV and Faculty Science Ltd; JR has received support for travel and accommodation from BTOG, ERS, CRUK, EORTC, ESMO and NCRI; RT has received honoraria from Olympus Medical; DRB has grant from NHS Digital and had received honoraria from MSD and AstraZeneca; DC, FM and JW are Optellum Ltd employees and have share options in Optellum Ltd. The remaining authors declare no conflicts of interests.

Word count: 3443

For peer review only

For peer review only - http://bmjopen.bmj.com/site/about/guidelines.xhtml

Reporting checklist for protocol of a clinical trial.

Based on the SPIRIT guidelines.

Instructions to authors

Complete this checklist by entering the page numbers from your manuscript where readers will find each of the items listed below.

Your article may not currently address all the items on the checklist. Please modify your text to include the missing information. If you are certain that an item does not apply, please write "n/a" and provide a short explanation.

Upload your completed checklist as an extra file when you submit to a journal.

In your methods section, say that you used the SPIRITreporting guidelines, and cite them as:

Chan A-W, Tetzlaff JM, Gøtzsche PC, Altman DG, Mann H, Berlin J, Dickersin K, Hróbjartsson A, Schulz KF, Parulekar WR, Krleža-Jerić K, Laupacis A, Moher D. SPIRIT 2013 Explanation and Elaboration: Guidance for protocols of clinical trials. BMJ. 2013;346:e7586

		Reporting Item	Page Number
**Administrative information**			
Title	#1	Descriptive title identifying the study design, population, interventions, and, if applicable, trial acronym	1

For peer review only

Trial registration	#2a	Trial identifier and registry name. If not yet registered, name of intended registry	2
Trial registration: data set	#2b	All items from the World Health Organization Trial Registration Data Set	2
Protocol version	#3	Date and version identifier	N/A
Funding	#4	Sources and types of financial, material, and other support	11
Roles and responsibilities: contributorship	#5a	Names, affiliations, and roles of protocol contributors	1
Roles and responsibilities: sponsor contact information	#5b	Name and contact information for the trial sponsor	11
Roles and responsibilities: sponsor and funder	#5c	Role of study sponsor and funders, if any, in study design; collection, management, analysis, and interpretation of data; writing of the report; and the decision to submit the report for publication, including whether they will have ultimate authority over any of these activities	11
Roles and responsibilities: committees	#5d	Composition, roles, and responsibilities of the coordinating centre, steering committee, endpoint adjudication committee, data management team, and	11

For peer review only

		other individuals or groups overseeing the trial, if applicable (see Item 21a for data monitoring committee)	
**Introduction**			
Background and rationale	#6a	Description of research question and justification for undertaking the trial, including summary of relevant studies (published and unpublished) examining benefits and harms for each intervention	3
Background and rationale: choice of comparators	#6b	Explanation for choice of comparators	3
Objectives	#7	Specific objectives or hypotheses	3
Trial design	#8	Description of trial design including type of trial (eg, parallel group, crossover, factorial, single group), allocation ratio, and framework (eg, superiority, equivalence, non-inferiority, exploratory)	3
**Methods: Participants, interventions, and outcomes**			
Study setting	#9	Description of study settings (eg, community clinic, academic hospital) and list of countries where data will be collected. Reference to where list of study sites can be obtained	4

For peer review only

Eligibility criteria	#10	Inclusion and exclusion criteria for participants. If applicable, eligibility criteria for study centres and individuals who will perform the interventions (eg, surgeons, psychotherapists)	4
Interventions: description	#11a	Interventions for each group with sufficient detail to allow replication, including how and when they will be administered	4-8
Interventions: modifications	#11b	Criteria for discontinuing or modifying allocated interventions for a given trial participant (eg, drug dose change in response to harms, participant request, or improving / worsening disease)	4-8
Interventions: adherance	#11c	Strategies to improve adherence to intervention protocols, and any procedures for monitoring adherence (eg, drug tablet return; laboratory tests)	4-8
Interventions: concomitant care	#11d	Relevant concomitant care and interventions that are permitted or prohibited during the trial	4-8
Outcomes	#12	Primary, secondary, and other outcomes, including the specific measurement variable (eg, systolic blood pressure), analysis metric (eg, change from baseline, final value, time to event), method of aggregation (eg, median, proportion), and time point for each outcome. Explanation of the clinical relevance of chosen efficacy and harm outcomes is strongly recommended	7/8

For peer review only

Participant timeline	#13	Time schedule of enrolment, interventions (including any run-ins and washouts), assessments, and visits for participants. A schematic diagram is highly recommended (see Figure)	4-8
Sample size	#14	Estimated number of participants needed to achieve study objectives and how it was determined, including clinical and statistical assumptions supporting any sample size calculations	8-9
Recruitment	#15	Strategies for achieving adequate participant enrolment to reach target sample size	4-9
**Methods: Assignment of interventions (for controlled trials)**			
Allocation: sequence generation	#16a	Method of generating the allocation sequence (eg, computer-generated random numbers), and list of any factors for stratification. To reduce predictability of a random sequence, details of any planned restriction (eg, blocking) should be provided in a separate document that is unavailable to those who enrol participants or assign interventions	N/A
Allocation concealment mechanism	#16b	Mechanism of implementing the allocation sequence (eg, central telephone; sequentially numbered, opaque,	N/A

For peer review only

		sealed envelopes), describing any steps to conceal the sequence until interventions are assigned	
Allocation: implementation	#16c	Who will generate the allocation sequence, who will enrol participants, and who will assign participants to interventions	N/A
Blinding (masking)	#17a	Who will be blinded after assignment to interventions (eg, trial participants, care providers, outcome assessors, data analysts), and how	N/A
Blinding (masking): emergency unblinding	#17b	If blinded, circumstances under which unblinding is permissible, and procedure for revealing a participant's allocated intervention during the trial	N/A
**Methods: Data collection, management, and analysis**			
Data collection plan	#18a	Plans for assessment and collection of outcome, baseline, and other trial data, including any related processes to promote data quality (eg, duplicate measurements, training of assessors) and a description of study instruments (eg, questionnaires, laboratory tests) along with their reliability and validity, if known. Reference to where data collection forms can be found, if not in the protocol	4-9

For peer review only

Data collection plan: retention	#18b	Plans to promote participant retention and complete follow-up, including list of any outcome data to be collected for participants who discontinue or deviate from intervention protocols	4-9
Data management	#19	Plans for data entry, coding, security, and storage, including any related processes to promote data quality (eg, double data entry; range checks for data values). Reference to where details of data management procedures can be found, if not in the protocol	4-9
Statistics: outcomes	#20a	Statistical methods for analysing primary and secondary outcomes. Reference to where other details of the statistical analysis plan can be found, if not in the protocol	8-9
Statistics: additional analyses	#20b	Methods for any additional analyses (eg, subgroup and adjusted analyses)	8-9
Statistics: analysis population and missing data	#20c	Definition of analysis population relating to protocol non-adherence (eg, as randomised analysis), and any statistical methods to handle missing data (eg, multiple imputation)	8-9
**Methods: Monitoring**			
Data monitoring: formal committee	#21a	Composition of data monitoring committee (DMC); summary of its role and reporting structure; statement of whether it is independent from the sponsor and competing interests; and reference to where further	9

For peer review only

		details about its charter can be found, if not in the protocol. Alternatively, an explanation of why a DMC is not needed	
Data monitoring: interim analysis	#21b	Description of any interim analyses and stopping guidelines, including who will have access to these interim results and make the final decision to terminate the trial	10
Harms	#22	Plans for collecting, assessing, reporting, and managing solicited and spontaneously reported adverse events and other unintended effects of trial interventions or trial conduct	10
Auditing	#23	Frequency and procedures for auditing trial conduct, if any, and whether the process will be independent from investigators and the sponsor	
**Ethics and dissemination**			
Research ethics approval	#24	Plans for seeking research ethics committee / institutional review board (REC / IRB) approval	9
Protocol amendments	#25	Plans for communicating important protocol modifications (eg, changes to eligibility criteria, outcomes, analyses) to relevant parties (eg, investigators, REC / IRBs, trial participants, trial registries, journals, regulators)	N/A

For peer review only

Consent or assent	#26a	Who will obtain informed consent or assent from potential trial participants or authorised surrogates, and how (see Item 32)	4-9
Consent or assent: ancillary studies	#26b	Additional consent provisions for collection and use of participant data and biological specimens in ancillary studies, if applicable	N/A
Confidentiality	#27	How personal information about potential and enrolled participants will be collected, shared, and maintained in order to protect confidentiality before, during, and after the trial	4-9
Declaration of interests	#28	Financial and other competing interests for principal investigators for the overall trial and each study site	11
Data access	#29	Statement of who will have access to the final trial dataset, and disclosure of contractual agreements that limit such access for investigators	N/A
Ancillary and post trial care	#30	Provisions, if any, for ancillary and post-trial care, and for compensation to those who suffer harm from trial participation	10
Dissemination policy: trial results	#31a	Plans for investigators and sponsor to communicate trial results to participants, healthcare professionals, the public, and other relevant groups (eg, via publication, reporting in results databases, or other data sharing arrangements), including any publication restrictions	10

For peer review only

Dissemination policy: authorship	#31b	Authorship eligibility guidelines and any intended use of professional writers	11
Dissemination policy: reproducible research	#31c	Plans, if any, for granting public access to the full protocol, participant-level dataset, and statistical code	N/A

Appendices

Informed consent materials	#32	Model consent form and other related documentation given to participants and authorised surrogates	N/A
Biological specimens	#33	Plans for collection, laboratory evaluation, and storage of biological specimens for genetic or molecular analysis in the current trial and for future use in ancillary studies, if applicable	N/A

None The SPIRIT Explanation and Elaboration paper is distributed under the terms of the Creative Commons Attribution License CC-BY-NC. This checklist can be completed online using https://www.goodreports.org/, a tool made by the EQUATOR Network in collaboration with Penelope.ai

For peer review only

BMJ Open

Determining the impact of an artificial intelligence tool on the management of pulmonary nodules detected incidentally on computed tomography (DOLCE) study protocol: a prospective, non-interventional multi-centre study

Journal:	*BMJ Open*
Manuscript ID	bmjopen-2023-077747.R1
Article Type:	Protocol
Date Submitted by the Author:	29-Sep-2023
Complete List of Authors:	O'Dowd, Emma; Nottingham University Hospitals NHS Trust<br>Berovic, Marko; King's College Hospital NHS Foundation Trust<br>CALLISTER, Matthew; Leeds Teaching Hospitals NHS Trust<br>Chalitsios, Christos ; University of Nottingham<br>Chopra, Disha; Optellum Ltd<br>Das, Indrajeet; University Hospitals of Leicester NHS Trust<br>Draper, Adrian; St George's Hospital, Respiratory Medicine<br>Garner, Justin; Royal Brompton and Harefield Hospitals<br>Gleeson, Fergus; Oxford University Hospitals NHS Foundation Trust<br>Janes, Sam; University College London<br>Kennedy, Martyn; Leeds Teaching Hospitals NHS Trust,<br>Lee, Richard; Royal Marsden Hospital NHS Trust<br>Mauri, Fabrizio; Optellum Ltd<br>McKeever, Tricia; University of Nottingham<br>McNulty, William; King's College Hospital NHS Foundation Trust<br>Murray, James; Royal Free London NHS Foundation Trust<br>Nair, Arjun; University College Hospital<br>Park, John; Oxford University Hospitals NHS Foundation Trust<br>Rawlinson, Janette; National Cancer Research Institute, Consumer forum, NCRI CSG (lung) subgroup, BTOG Steering Committee, NHSE CEG;<br>Sago, Gurdeep; University of Newcastle upon Tyne<br>Scarsbrook, Andrew; Leeds Teaching Hospitals NHS Trust<br>Shah, Pallav; Royal Brompton and Harefield NHS Foundation Trust<br>Sudhir, Rajini; University Hospitals of Leicester NHS Trust<br>Talwar, Ambika; Oxford University Hospitals NHS Foundation Trust<br>Thakrar, Ricky; University College London Hospitals NHS Foundation Trust<br>Watkins, Johnathan; Optellum Ltd<br>Baldwin, David; Nottingham University Hospitals NHS Trust
**Primary Subject Heading**:	Respiratory medicine
Secondary Subject Heading:	Diagnostics, Oncology, Radiology and imaging, Respiratory medicine
Keywords:	Clinical Trial, Chest imaging < RADIOLOGY & IMAGING, RADIOLOGY &

	IMAGING, Computed tomography < RADIOLOGY & IMAGING, Respiratory tract tumours < THORACIC MEDICINE

SCHOLARONE™ Manuscripts

Title

Determining the impact of an artificial intelligence tool on the management of pulmonary nodules detected incidentally on computed tomography (DOLCE) study protocol: a prospective, non-interventional multi-centre study.

Emma L O'Dowd[1], Marko Berovic[2], Matthew Callister[3], Christos V. Chalitsios[4], Disha Chopra[5], Indrajeet Das[6], Adrian Draper[7], Justin L Garner[8], Fergus Gleeson[9], Sam Janes[10], Martyn Kennedy[3], Richard Lee[11], Fabrizio Mauri[5], Tricia McKeever[4], William McNulty[2], James Murray[12], Arjun Nair[13], John Park[9], Janette Rawlinson[14], Gurdeep S Sagoo[15], Andrew Scarsbrook[3], Pallav L Shah[8], Rajini Sudhir[6], Ambika Talwar[9], Ricky Thakrar[13], Johnathan Watkins[5], David R Baldwin[1]

1. Nottingham University Hospitals NHS Trust, Nottingham, NG5 1PB

2. King's College Hospital NHS Foundation Trust, London SE5 9RS

3. Leeds Teaching Hospitals NHS Trust, Leeds LS9 7TF

4. University of Nottingham, Nottingham, NG5 1PB

5. Optellum Ltd, Oxford Centre For Innovation, New Road, Oxford, England, OX1 1BY

6. University Hospitals of Leicester NHS Trust, Leicester, Leicester, LE3 9QP

7. St George's University Hospitals NHS Foundation Trust, London, SW17 0QT

8. Royal Brompton and Harefield hospitals, London SW3 6NP

9. Oxford University Hospitals NHS Trust, Oxford, UK

10. Lungs for Living Research Centre, University College London, London, UK

11. The Royal Marsden NHS Foundation Trust, London SW3 6JJ

12. Royal Free London NHS Foundation Trust, London NW3 2QG

13. University College London Hospitals NHS Foundation Trust, London

14. Patient representative, National Cancer Research Institute (NCRI), Consumer forum, NCRI CSG (Lung) Subgroup, BTOG Steering Committee, NHSE CEG, UK

15. University of Newcastle, Newcastle upon Tyne

Corresponding author: Emma L O'Dowd (emma.o'dowd@nottingham.ac.uk)

For peer review only - http://bmjopen.bmj.com/site/about/guidelines.xhtml

Abstract

Introduction

In a small percentage of patients, pulmonary nodules found on computed tomography (CT) scans are early lung cancers. Lung cancer detected at an early stage has a much better prognosis. The British Thoracic Society (BTS) guideline on managing pulmonary nodules recommends using multivariable malignancy risk prediction models to assist in management. Whilst these guidelines seem to be effective in clinical practice, recent data suggest that artificial intelligence (AI)-based malignant-nodule prediction solutions might outperform existing models.

Methods and analysis

This study is a prospective, observational multicentre study to assess the clinical utility of an AI-assisted CT-based lung cancer prediction tool (LCP) for managing incidental solid and part solid pulmonary nodule patients versus standard care. Two thousand patients will be recruited from 12 different UK hospitals. The primary outcome is the difference between standard care and LCP-guided care in terms of the rate of benign-nodules and cancer patients discharged straight after the assessment of the baseline CT scan. Secondary outcomes investigate adherence to clinical guidelines, other measures of changes to clinical management, patient outcomes, and cost-effectiveness.

Ethics and dissemination

This study has been reviewed and given a favourable opinion by the South Central - Oxford C Research Ethics Committee in UK (REC reference number: 22/SC/0142).

Study results will be available publicly following peer-reviewed publication in open-access journals. A Patient and Public Involvement (PPI) group's workshop is planned before the study results will be available to discuss best methods to disseminate the results. Study results will also be fed back to participating organisations to inform training and procurement activities.

Trial registration number

The study is registered in Clinicaltrials.gov, number: NCT05389774

Strengths and limitations

- This is the first prospective study examining the use of artificial intelligence (AI)-based computer-aided diagnosis (CADx) for pulmonary nodules
- This is also the first study to examine the clinical utility of an AI-based CADx tool for pulmonary nodule management
- The study is non-interventional in nature
- The analysis of some of the secondary objectives is exploratory due to potential sample size restrictions

Keywords

Clinical trial; chest imaging; radiology & imaging; computed tomography; respiratory tract tumours

For peer review only

For peer review only - http://bmjopen.bmj.com/site/about/guidelines.xhtml

Introduction

From 2017 to 2019, lung cancer caused 34,771 deaths in the UK per annum, more than prostate and bowel cancer combined (28,846), the next most common causes of cancer death. Over the same period, there were 24% more deaths in women from lung cancer than from breast malignancy (14,140 vs 11,415) [1]. This is because almost three-quarters of people with lung cancer present with stage III or IV disease when the prognosis is poor despite recent advances in systemic anti-cancer treatment. Lung cancer detected at an early stage, usually by thoracic computed tomography (CT), has a much better prognosis. Screening with low-dose CT thorax has recently been confirmed to reduce lung cancer and all-cause mortality [2–4]. Central to the success of screening is the accurate management of pulmonary nodules, which may represent early-stage lung cancer, but are far more often benign. Pulmonary nodules are also a common finding on CT scans done for other reasons. In some settings, this may yield more early lung cancers than screening. Identifying malignant pulmonary nodules on CT scans is the primary way to detect lung cancer at stage I, when the prognosis is best. By definition, isolated pulmonary nodules without other lung changes, are stage I if proven to be lung cancer. Currently, most nodules are detected outside screening programmes in the UK, providing an important rationale for this study to address the effective management of these incidentally detected nodules.

The British Thoracic Society (BTS) guideline on managing pulmonary nodules was published in 2015 [5]. This guidance was the first to recommend semi-automated volumetry as the standard for nodule measurement and the use of multivariable malignancy risk prediction models to assist in management. Whilst these guidelines seem to be effective in clinical practice [6], recent data suggest that artificial intelligence (AI)-based malignant-nodule prediction solutions appear to outperform existing multivariable models [7,8]. This could result in a considerable reduction in the number of follow-up CT, which is much needed in the NHS, where current and forecasted demand for radiology services exceeds capacity [9,10].

Optellum Ltd has developed and extensively validated an AI-based computer-aided diagnostic (CADx) tool for lung cancer prediction (LCP) in pulmonary nodules, which has UK Conformity Assessed marking under UK medical device regulations. The AI model underpinning the LCP is a convolutional neural network (CNN), referred to hereafter as the 'LCP-CNN'. LCP-CNN was derived and internally validated using data from diverse populations from the US and UK. The model was then externally validated in two independent cohorts of patients with incidentally detected pulmonary nodules, amounting to over 1,500 patients in aggregate [7,8]. The LCP-CNN demonstrated greater accuracy in these studies than the Brock and Mayo malignancy risk models (used in managing incidentally detected nodules in the UK and US, respectively). The product is provided within the "Virtual Nodule Clinic" (VNC), a software device used in the tracking, assessment and characterization of incidentally detected pulmonary nodules.

The study aims to assess this new AI tool in a prospective real-world setting in the UK. This will be done by calculating malignancy risk using LCP and measuring how management would differ if it were governed by the risk provided. The aim is to provide evidence as to whether LCP should be the new standard of care in pulmonary nodule management.

For peer review only - http://bmjopen.bmj.com/site/about/guidelines.xhtml

Methods and analysis

The study acronym is "DOLCE" (Determining the impact of Optellum's LCP artificial intelligence solution on service utilisation, health Economics and patient outcomes).

Design and setting

DOLCE is a multi-centre prospective observational cohort study recruiting patients with 5-30 mm solid and part-solid pulmonary nodules incidentally detected on thoracic CT scans performed as part of routine practice. The study aims to recruit 2,000 patients from 12 centres.

Patients will be asked to consent to and be enrolled at twelve acute NHS hospital trusts in England. A recruitment period of one year is planned, with one year of follow-up to collect data and ascertain the ground-truth diagnosis of recruited patients.

The start date was March 2023 with the planned recruitment end date being February 2024. With one year of follow up, this will mean a planned end date of February 2025.

Clinical pathway

This is a non-interventional study. There will be no anticipated change to the standard of care the recruited patients receive throughout the study's duration. The potential impact of AI-modified management will be assessed by comparing routine clinical management with that which would have been conditional upon the LCP risk score. The scores will be provided to clinicians after routine management decisions have been made, either during the same MDT meeting or at a separate research meeting. A hypothetical clinical decision based on the LCP score will then be recorded.

Study population

Patients who have undergone CT that has detected a pulmonary nodule and was performed for a reason other than suspicion of lung cancer will be reviewed for study eligibility (Table 1).

Table 1: Patient eligibility criteria

**Inclusion criteria**
Patients aged 35 years or over
Patients with a baseline CT study with at least one incidentally detected solid or part-solid pulmonary nodule that is 5-30mm in maximum axial diameter for the whole lesion, measured using manual electronic callipers, and that is not fully calcified. The solid component of the part-solid nodule must be ≥ 80% of the total nodule size.
Patients with a baseline CT study which includes at least one series with acquisition parameters which meet VNC requirements
The patient has a baseline CT study that includes at least one series that comprises at least one full-inspiration breath-hold scan without a high degree of contrast media and does not exhibit quality issues (e.g., motion artefacts)
**Exclusion criteria**
Patients who received a diagnosis of cancer in the last 5 years

For peer review only - http://bmjopen.bmj.com/site/about/guidelines.xhtml

Patients who have thoracic implants that adversely impact the imaging appearances of the nodule
Patients who have more than five reported pulmonary nodules of any size or type, excluding fully calcified nodules (a marker of prior granulomatous infection or metastatic lung cancer)
Patients who have one or more additional nodules that are already undergoing follow-up according to pulmonary nodule management standard care
Patients who have one or more additional nodules that are pure ground glass opacity (GGO) of 5mm in maximum axial diameter for the whole lesion measured using manual electronic callipers
Patients who have one or more additional nodules >30mm in maximum axial diameter for the whole lesion measured using manual electronic callipers.
Patients with part-solid nodules where the solid component is < 80% of the total nodule size.

AI model

The output of the LCP is a risk score that is an integer from 1 to 10 and forms part of Optellum's VNC patient management software. A score of 1 indicates the nodule is highly likely to be benign, while a score of 10 is most likely to be malignant. The AI model underpinning the LCP is the LCP-CNN, the raw score available to researchers as a continuous value from 0 to 100, as previously reported. LCP-CNN was derived and internally validated using data from diverse populations from the US and UK. The model was then externally validated in two independent cohorts of patients with incidentally detected pulmonary nodules, amounting to over 1,500 patients in aggregate [7,8].

In this study, the LCP will be compared to standard care in three ways:

1. LCP as an integer (1-10) risk score to aid physician decision-making (physician guided by AI score)
2. LCP-CNN alone as a threshold-based rule-out recommendation (AI binary decision to discharge or not)
3. LCP-CNN combined with a threshold-based rule-out recommendation to aid physician decision-making (physician guided by AI with binary recommendation to discharge or not)

Data collection

Study teams at the twelve NHS Trusts from which patients will be recruited will identify eligible patients and obtain informed consent. Consent will be obtained verbally (for patients who would not normally come on-site as part of standard care) or via written consent. Consent will be sought to analyse standard-care CT scans, associated clinical meta-data collection, and the health economics assessment.

Once a patient has given consent and enrolled into the study, their baseline CT scan will be sent to VNC. Authorised research staff will then log into VNC, select the nodules of interest, and record the LCP score for that nodule. The local standard nodule service decision-makers will provide a standard-care decision for eligible, consented patients via their usual nodule

For peer review only

For peer review only - http://bmjopen.bmj.com/site/about/guidelines.xhtml

management pathway. The standard-care decision for eligible consented patients will then be recorded in an electronic Case Report Form (eCRF) without knowing the LCP score.

To assess LCP under scenario #1 as described under *AI model*, the clinical team will thereafter be given the LCP score and relevant patient data in the nodule MDT or as part of a separate research meeting. At this point, the clinical team should remain blinded to the outcome of any actual-care investigation (e.g., PET/CT scan, biopsy etc.). Their decision on the next management step, had they been guided by the LCP score, will then be recorded in the eCRF. The clinical team will have the option to modify management at this point. Any modification of management will be recorded in the eCRF.

Subsequent follow-up images for each patient will be sent to the VNC over the course of their nodule follow-up, and the final nodule diagnosis will be recorded. Since standard patient care can sometimes deviate from BTS guidelines, all participating sites will also collect information on patient and nodule characteristics necessary to follow the BTS guidelines. In this way, AI-guided management can be compared with BTS-compliant management. Other data points necessary to evaluate service utilisation (e.g. additional scans, biopsies, and treatments for lung nodules or lung cancers) will also be recorded in the eCRF.

In line with SPIRIT-AI guidelines, performance errors that would have happened if the LCP or LCP-guided decision making were part of standard care will be analysed and reported as outcome measures for the primary objective of this study.

Evaluation of LCP as threshold-based tests (scenarios #2 and #3 as described under *AI model*), which require access to LCP-CNN values, will be performed as a batch at the end of the study follow-up period. The threshold will be defined prospectively based on values from training datasets of similar patient populations. The use of the recommendation with physicians making the decision will be evaluated via a separate research meeting at the end of the 12-month follow-up period.

For cost-effectiveness modelling, quality of life data will be collected by inviting patients to complete an EQ-5D-5L quality of life questionnaire at two-time points: a) as close as possible to the baseline CT scan on which their pulmonary nodule was detected; and b) at a 1-year timepoint afterwards. A participant diary about primary care usage will also be collected at the one-year mark.

Ground truth diagnosis

Patients will be classified into whether they have benign or malignant nodules as follows:

1. Benign-nodule patients:
    a. By resolution or stability of all qualifying incidental pulmonary nodules as determined from volumetry-based measurement on imaging follow-up over 12 months
    b. By histopathology from biopsy or surgery of all qualifying incidental pulmonary nodules (within 12 months of baseline CT scan)
2. Malignant-nodule patients:
    a. By histopathology from biopsy or surgery (within 12 months of baseline CT scan) of at least one qualifying incidental pulmonary nodule
3. Probable benign-nodule patients:

For peer review only - http://bmjopen.bmj.com/site/about/guidelines.xhtml

a. By resolution, stability, or shrinkage of qualifying incidental pulmonary nodules that are not in ground-truth outcomes #1 or #2 above as determined from volumetry-based measurement on imaging follow-up conducted between 3-12 months after the baseline CT scan
b. By resolution, stability, or shrinkage of qualifying incidental pulmonary nodules that are not in ground-truth outcomes 1 or 2 above as determined from diameter-based measurement on imaging follow-up conducted 12 months after the baseline CT scan (supplemented with a volume-based measurement of the nodule that will be performed for research purposes)

4. Probable malignant-nodule patients:
    a. By clinical diagnosis of lung cancer, in whom a biopsy or resection is not possible, based on high clinical probability and who are registered at a lung cancer MDT/and/or receive non-surgical treatment (SABR/ radiotherapy/ microwave ablation)

Volumetry-based measurement of nodule stability – and hence a benign diagnosis – will be satisfied if the lung nodule volume on the follow-up CT scan was <125% of the lung nodule volume on the baseline CT scan.

If the standard care at a particular site does not incorporate volumetry-based measurements, the volumetric measurement will be applied as part of the research study to the relevant follow-up CT scans.

Any lung nodule patients who do not fall into either of the two ground-truth outcomes 1 or 2 above will be considered to have an indeterminate diagnosis at the time of analysis and will be analysed with and without the inclusion of the ground-truth outcomes in 3 and 4.

Outcome measures

The outcome measures for the primary and secondary objectives are outlined in Table 2.

Table 2: Study objectives and outcome measures

Objective	Outcome measure
**Primary**:<br>Determine the potential effect of the LCP on discharge	The measured difference between standard care and the three LCP-based options described under *AI model* for:<br>• Number and percentage of cancer patients discharged (straight after assessment of the baseline scan)<br>• Number and percentage of benign-nodule patients discharged (straight after assessment of the baseline scan)
**Secondary**:<br>Determine the potential effect of the LCP on possible	The measured difference between standard care and the three LCP-based options described under *AI model*:<br>• Number and percentage of patients for whom a validated risk model (Brock or LCP) is used to guide the next clinical management step (i.e., counting the instances where Brock is not used or where LCP is not possible to compute, or it is ignored)

For peer review only - http://bmjopen.bmj.com/site/about/guidelines.xhtml

adherence to clinical guidelines	
**Secondary**:<br>Determine the potential effect of the LCP on overall clinical management, as well as scan and procedure utilisation	Number and percentage of cancer patients for whom there would have been a change in clinical management according to each of the three LCP-based options described under *AI model* compared with actual care (correctly for more aggressive management and incorrectly for less aggressive management).<br>Number and percentage of benign-nodule patients for whom there would have been a change in clinical management according to each of the three LCP-based options described under *AI model* compared with actual care (incorrectly by more aggressive management and correctly for less aggressive management).<br>The measured difference between standard care and the three LCP-based options described under *AI model* for:<br>• Number and percentage of CT scans and PET/CT scans performed on benign-nodule patients<br>• Number and percentage of non-surgical biopsies performed on benign-nodule patients<br>• Number and percentage of surgical excisions on benign-nodule patients
**Secondary**:<br>Determine the potential effect of LCP-guided care versus standard care on patient outcomes	The measured difference between standard care and the three LCP-based options described under *AI model* for:<br>• Number and percentage of thoracic, respiratory, or vascular events related to biopsies or surgical excisions for lung nodules or suspected lung cancer occurring within 30 days of the procedure on benign-nodule patients<br>• Number and percentage of lung cancers stratified by stage<br>• Time in days between nodule detection and lung cancer diagnosis
**Secondary**:<br>Determine the potential health-economic effect of LCP-guided care versus standard care	The measured difference between standard care and the three LCP-based options described under *AI model* for:<br>• The composite standardised GBP costs of all healthcare-related activity for lung nodules or suspected lung cancer<br>• Health-Related Quality of Life (using EQ5D5L to generate QALYs)<br>• Estimate cost-effectiveness (Incremental costs per QALY gained)

Statistical analysis

Sample size

For calculations relating to the primary objectives of a measured difference between standard care and LCP for:

For peer review only

For peer review only - http://bmjopen.bmj.com/site/about/guidelines.xhtml

- Number and percentage of cancer patients discharged: results from thresholds defined for discharge in a previous study are used [7]. Based on previous data within the cancer group, assuming for the discordant pairs that the Brock model threshold (representing the standard of care) identified 0.4% of cancers for which the LCP threshold would have recommended discharge, and the LCP threshold correctly identified 2.6% of cancers for which the Brock model threshold would have recommended discharge, then using McNemar's test with 90% power and a significance level of 0.05, 659 participants would be needed. However, with a sample size of 2000, there is 90% power to detect a difference between the methods if the percentages were as small as 1.0% and 2.3%, respectively.
- Number and percentage of benign-nodule patients discharged: again, results from thresholds defined for discharge in a previous study are used [7]. Based on previous data within the benign group, assuming for the discordant pairs that 17.0% of benign nodules are recommended for discharge by the LCP threshold but not by the Brock model threshold. In comparison, 13.5% are recommended for discharge by the Brock model threshold but not by the LCP threshold; there is 80% power to detect a significant difference between the two tests with 2,026 individuals using McNemar's test. This equates to an odds ratio of 1.26

Sample size calculations were performed using R statistical software (version 4.1.2; R Foundation for Statistical Computing, Vienna, Austria).

Statistical analysis

Statisticians will carry out all analyses. For each outcome measure, the following will be reported:

- The number of participants included in the analysis, stratified by the relevant group
- Mean or median (SD or IQR) for continuous outcomes, and numbers and proportions (percentage) for binary outcomes
- An effect size (odds ratio) with a 95% confidence interval
- A two-sided p-value

For both primary objectives, measured differences between standard care and the LCP options for cancer patients diagnosed and benign-nodule patients discharged will be analysed using conditional logistic regression. Statistical significance will be assessed with a Wald test. Secondary outcomes will be described and analysed according to their distribution. Correction for multiple testing will be performed.

Health economics analysis

The economic analysis will estimate the expected costs and expected QALYs gained from standard care, LCP, and LCP-guided care over patient lifetime from the perspective of the UK NHS. Incremental analyses will identify the most cost-effective permutation with exploratory analyses undertaken to determine the uncertainty around measures of cost-effectiveness. Costs will be calculated as quantities of resource use multiple by appropriate unit costs (e.g. NHS Reference Costs) and will be specific to a year to be determined at time of analysis. A HEAP (health economics analysis plan) will be produced prior to undertaking the analysis at the end of the study patient follow-up period.

For peer review only - http://bmjopen.bmj.com/site/about/guidelines.xhtml

Patient and public involvement

An extensive programme of patient and public involvement (PPI) input was arranged to provide input into the study objectives, design, materials and governance and planned to disseminate study results.

Specifically, a PPI representative, who sits on the Trial Management Group, provided input from study conception to developing study materials. This guidance and support will continue during the management and conduct of the study. An independent PPI contributor is a member of the trial steering committee (TSC). Further input was gathered from a group of PPI contributors selected from across the country with varying experiences of lung nodules, cancer or none from a patient and/or carer perspective during facilitated virtual workshops.

Input received to date has ranged from considerations of healthcare inequalities and other biases, awareness of AI generally and in healthcare, methods of and materials for participant recruitment and communication, and the design of any data collected during the study.

Ethics and dissemination

This study has been reviewed and given a favourable opinion by the South Central - Oxford C Research Ethics Committee in UK (REC reference number: 22/SC/0142).

Given the study's observational nature, we do not envisage any elevation to the harms or the risk of harm for patients. The sponsor and Chief Investigator have conducted a full risk assessment.

Patients will be asked to give their consent to participate in the study (including the analysis of standard-care CT scans, associated data collection and the healthcare economics assessment) by telephone or face-to-face when attending routine clinic appointments. No additional patient visits, scans or procedures are planned for the study. Patients not wishing to participate in the health economics assessment may still participate in the study's CT imaging analysis.

The findings of this study will be shared with the funder (NHS Accelerated Access Collaborative). Results will be presented at academic and clinical conferences. The study will result in at least one peer-reviewed publication of the findings in an international medical journal.

References

1 Cancer Research UK. Cancer mortality for common cancers. 2022.https://www.cancerresearchuk.org/health-professional/cancer-statistics/mortality/common-cancers-compared

2 Lindholt JS, Søgaard R. Lung-Cancer Screening and the NELSON Trial. *N Engl J Med* 2020;**382**:2164. doi:10.1056/NEJMc2004224

3 de Koning HJ, van der Aalst CM, de Jong PA, *et al.* Reduced Lung-Cancer Mortality with Volume CT Screening in a Randomized Trial. *N Engl J Med* 2020;**382**:503–13. doi:10.1056/NEJMoa1911793

For peer review only - http://bmjopen.bmj.com/site/about/guidelines.xhtml

4 National Lung Screening Trial Research Team, Aberle DR, Adams AM, *et al.* Reduced lung-cancer mortality with low-dose computed tomographic screening. *N Engl J Med* 2011;**365**:395–409. doi:10.1056/NEJMoa1102873

5 Callister MEJ, Baldwin DR, Akram AR, *et al.* British Thoracic Society guidelines for the investigation and management of pulmonary nodules. *Thorax* 2015;**70 Suppl 2**:ii1–54. doi:10.1136/thoraxjnl-2015-207168

6 Balata H, Ruparel M, O'Dowd E, *et al.* Analysis of the baseline performance of five UK lung cancer screening programmes. *Lung Cancer* 2021;**161**:136–40. doi:10.1016/j.lungcan.2021.09.012

7 Baldwin DR, Gustafson J, Pickup L, *et al.* External validation of a convolutional neural network artificial intelligence tool to predict malignancy in pulmonary nodules. *Thorax* 2020;**75**:306–12. doi:10.1136/thoraxjnl-2019-214104

8 Massion PP, Antic S, Ather S, *et al.* Assessing the Accuracy of a Deep Learning Method to Risk Stratify Indeterminate Pulmonary Nodules. *Am J Respir Crit Care Med* 2020;**202**:241–9. doi:10.1164/rccm.201903-0505OC

9 The Royal College of Radiologists. RCR Clinical radiology census report 2021. 2021.

10 Maskell G. Why does demand for medical imaging keep rising? *BMJ* 2022;:o2614. doi:10.1136/bmj.o2614

Authors' contributions

All authors (EOD, DRB, JW, FM, MB, MC, CC, DC, ID, AD, JG, FG, SJ, MK, RL, TM, WM, JM, AN, JP, JR, GS, AS, PS, RS, AT, RT) contributed to the development and set up of the study. EOD, DRB, JW, FM wrote the manuscript, all authors (EOD, DRB, JW, FM, MB, MC, CC, DC, ID, AD, JG, FG, SJ, MK, RL, TM, WM, JM, AN, JP, JR, GS, AS, PS, RS, AT, RT) contributed to, reviewed and approved the final version of the manuscript.

Funding statement

This work is supported NHS Accelerated Access Collaborative. Grant number AI_AWARD02266. Sponsor organisation is Nottingham University Hospitals NHS Trust. The sponsor organisation and funder have had no role in study design.

Competing interests

FG reports grant funding from NIHR and Innovate UK, consultancy fees from Polarean Ltd and has shares in Optellum Ltd; SJ has received grant funding from GRAIL Ltd, consultancy fees/ honoraria from AstraZeneca and Chiesi, stock options in Optellum Ltd and his spouse is employed by AstraZeneca; RL is funded by the Royal Marsden NIHR BRC, Royal Marsden Cancer charity and SBRI (including QURE.AI). RL's institution receives compensation for time spent in a secondment role for the lung health check program and as a National Specialty Lead for the National Institute of Health and Care Research. He has received research funding from CRUK, Innovate UK (co-funded by GE Healthcare, Roche and Optellum), SBRI, RM Partners Cancer Alliance and NIHR (co-applicant in grants with Optellum). He has received

For peer review only - http://bmjopen.bmj.com/site/about/guidelines.xhtml

honoraria from CRUK; WM has received honoraria from Amgen; AN declares grants from Department of Health's NIHR Biomedical Research Centre's funding scheme and GRAIL, consulting fees from Aidence BV, Faculty Science LTD and MSD; Support for attending meetings from Takeda Limited; Advisory Board participation for Aidence BV and Faculty Science Ltd; JR has received support for travel and accommodation from BTOG, ERS, CRUK, EORTC, ESMO and NCRI; RT has received honoraria from Olympus Medical; DRB has grant from NHS Digital and had received honoraria from MSD and AstraZeneca; DC, FM and JW are Optellum Ltd employees and have share options in Optellum Ltd. The remaining authors declare no conflicts of interests.

Word count: 2686

For peer review only

For peer review only - http://bmjopen.bmj.com/site/about/guidelines.xhtml

SPIRIT-AI

SPIRIT-AI Checklist: Recommended items to address in a protocol and related documents for clinical trials evaluating AI interventions

Section	SPIRIT 2013 Item[a]		SPIRIT-AI Item		Addressed on Page No [b]
Administrative Information					
Title	1	Descriptive title identifying the study design, population, interventions, and, if applicable, trial acronym	SPIRIT-AI 1(i) Elaboration	Indicate that the intervention involves artificial intelligence / machine learning and specify the type of model.	1
			SPIRIT-AI 1(ii) Elaboration	Specify the intended use of the AI intervention.	3
Trial registration	2a	Trial identifier and registry name. If not yet registered, name of intended registry	2		
	2b	All items from the World Health Organization Trial Registration Data Set	2		
Protocol version	3	Date and version identifier	NA		
Funding	4	Sources and types of financial, material, and other support	11		
Roles and responsibilities	5a	Names, affiliations, and roles of protocol contributors	1		
	5b	Name and contact information for the trial sponsor	11		
	5c	Role of study sponsor and funders, if any, in study design; collection, management, analysis, and interpretation of data; writing of the report; and the decision to submit the report for publication, including whether they will have ultimate authority over any of these activities	11		
	5d	Composition, roles, and responsibilities of the coordinating centre, steering committee, endpoint adjudication committee, data management team, and other individuals or groups overseeing the trial, if applicable (see Item 21a for data monitoring committee)	11		
Introduction					
Background and rationale	6a	Description of research question and justification for undertaking the trial, including summary of relevant studies (published and unpublished) examining benefits and harms for each intervention	SPIRIT-AI 6a (i) Extension	Explain the intended use of the AI intervention in the context of the clinical pathway, including its purpose and its intended users (e.g. healthcare professionals, patients, public).	3
			SPIRIT-AI 6a (ii) Extension	Describe any pre-existing evidence for the AI intervention.	3

Cite as:

For peer review only

SPIRIT-AI

	6b	Explanation for choice of comparators	3		
Objectives	7	Specific objectives or hypotheses	3		
Trial design	8	Description of trial design including type of trial (eg, parallel group, crossover, factorial, single group), allocation ratio, and framework (eg, superiority, equivalence, noninferiority, exploratory)	3		
Methods: Participants, Interventions and Outcomes					
Study setting	9	Description of study settings (eg, community clinic, academic hospital) and list of countries where data will be collected. Reference to where list of study sites can be obtained	SPIRIT-AI 9 Extension	Describe the onsite and offsite requirements needed to integrate the AI intervention into the trial setting.	5, 6
Eligibility criteria	10	Inclusion and exclusion criteria for participants. If applicable, eligibility criteria for study centres and individuals who will perform the interventions (eg, surgeons, psychotherapists)	SPIRIT-AI 10 (i) Elaboration	State the inclusion and exclusion criteria at the level of participants.	4, 5
			SPIRIT-AI 10 (ii) Extension	State the inclusion and exclusion criteria at the level of the input data.	4, 5
Interventions	11a	Interventions for each group with sufficient detail to allow replication, including how and when they will be administered	SPIRIT-AI 11a (i) Extension	State which version of the AI algorithm will be used.	5
			SPIRIT-AI 11a (ii) Extension	Specify the procedure for acquiring and selecting the input data for the AI intervention.	5
			SPIRIT-AI 11a (iii) Extension	Specify the procedure for assessing and handling poor quality or unavailable input data.	4-7
			SPIRIT-AI 11a (iv) Extension	Specify whether there is human-AI interaction in the handling of the input data, and what level of expertise is required for users.	4-7
			SPIRIT-AI 11a (v) Extension	Specify the output of the AI intervention.	4-7
			SPIRIT-AI 11a (vi) Extension	Explain the procedure for how the AI intervention's output will contribute to decision-making or other elements of clinical practice.	4-7
	11b	Criteria for discontinuing or modifying allocated interventions for a given trial participant (eg, drug dose change in response to harms, participant request, or improving/worsening disease)	4-8		
	11c	Strategies to improve adherence to intervention protocols, and any procedures for monitoring adherence (eg, drug tablet return, laboratory tests)	4-8		
	11d	Relevant concomitant care and interventions that are permitted or prohibited during the trial	4-8		
Outcomes	12	Primary, secondary, and other outcomes, including the specific measurement variable (eg, systolic blood pressure), analysis metric (eg, change from baseline, final value, time to event), method of aggregation (eg, median, proportion), and time point for each outcome. Explanation of the clinical relevance of chosen efficacy and harm outcomes is strongly recommended	7/8		

Cite as:

For peer review only - http://bmjopen.bmj.com/site/about/guidelines.xhtml

Participant timeline	13	Time schedule of enrolment, interventions (including any run-ins and washouts), assessments, and visits for participants. A schematic diagram is highly recommended (see Figure)	4-8		
Sample size	14	Estimated number of participants needed to achieve study objectives and how it was determined, including clinical and statistical assumptions supporting any sample size calculations	8-9		
Recruitment	15	Strategies for achieving adequate participant enrolment to reach target sample size	4-9		
Methods: Assignment of Interventions (For Controlled Trials)					
Sequence generation	16A	Method of generating the allocation sequence (eg, computer-generated random numbers), and list of any factors for stratification. To reduce predictability of a random sequence, details of any planned restriction (eg, blocking) should be provided in a separate document that is unavailable to those who enrol participants or assign interventions	NA		
Allocation concealment mechanism	16b	Mechanism of implementing the allocation sequence (eg, central telephone; sequentially numbered, opaque, sealed envelopes), describing any steps to conceal the sequence until interventions are assigned	NA		
Implementation	16c	Who will generate the allocation sequence, who will enrol participants, and who will assign participants to interventions	NA		
Blinding (masking)	17a	Who will be blinded after assignment to interventions (eg, trial participants, care providers, outcome assessors, data analysts), and how	NA		
	17b	If blinded, circumstances under which unblinding is permissible, and procedure for revealing a participant's allocated intervention during the trial	NA		
Methods: Data Collection, Management, And Analysis					
Data collection methods	18a	Plans for assessment and collection of outcome, baseline, and other trial data, including any related processes to promote data quality (eg, duplicate measurements, training of assessors) and a description of study instruments (eg, questionnaires, laboratory tests) along with their reliability and validity, if known. Reference to where data collection forms can be found, if not in the protocol	4-9		
	18b	Plans to promote participant retention and complete follow-up, including list of any outcome data to be collected for participants who discontinue or deviate from intervention protocols	4-9		
Data management	19	Plans for data entry, coding, security, and storage, including any related processes to promote data quality (eg, double data entry; range checks for data values). Reference to where details of data management procedures can be found, if not in the protocol	4-9		

Cite as:

For peer review only - http://bmjopen.bmj.com/site/about/guidelines.xhtml

SPIRIT-AI

Statistical methods	20a	Statistical methods for analysing primary and secondary outcomes. Reference to where other details of the statistical analysis plan can be found, if not in the protocol	8-9			
	20b	Methods for any additional analyses (eg, subgroup and adjusted analyses)	8-9			
	20c	Definition of analysis population relating to protocol non-adherence (eg, as randomised analysis), and any statistical methods to handle missing data (eg, multiple imputation)	8-9			
Methods: Monitoring						
Data monitoring	21a	Composition of data monitoring committee (DMC); summary of its role and reporting structure; statement of whether it is independent from the sponsor and competing interests; and reference to where further details about its charter can be found, if not in the protocol. Alternatively, an explanation of why a DMC is not needed	9-10			
	21b	Description of any interim analyses and stopping guidelines, including who will have access to these interim results and make the final decision to terminate the trial	10			
Harms	22	Plans for collecting, assessing, reporting, and managing solicited and spontaneously reported adverse events and other unintended effects of trial interventions or trial conduct		SPIRIT-AI 22 Extension	Specify any plans to identify and analyse performance errors. If there are no plans for this, explain why not.	5-10
Auditing	23	Frequency and procedures for auditing trial conduct, if any, and whether the process will be independent from investigators and the sponsor	10			
Ethics and Dissemination						
Research ethics approval	24	Plans for seeking research ethics committee/institutional review board (REC/IRB) approval	10			
Protocol amendments	25	Plans for communicating important protocol modifications (eg, changes to eligibility criteria, outcomes, analyses) to relevant parties (eg, investigators, REC/IRBs, trial participants, trial registries, journals, regulators)	9-10			
Consent or ascent	26a	Who will obtain informed consent or assent from potential trial participants or authorised surrogates, and how (see Item 32)	4-9			
	26b	Additional consent provisions for collection and use of participant data and biological specimens in ancillary studies, if applicable	NA			
Confidentiality	27	How personal information about potential and enrolled participants will be collected, shared, and maintained in order to protect confidentiality before, during, and after the trial	4-9			
Declaration of interests	28	Financial and other competing interests for principal investigators for the overall trial and each study site	11-12			

Cite as:

For peer review only

Access to data	29	Statement of who will have access to the final trial dataset, and disclosure of contractual agreements that limit such access for investigators	SPIRIT-AI 29 Extension	State whether and how the AI intervention and/or its code can be accessed, including any restrictions to access or re-use.	4-10
Ancillary and post-trial care	30	Provisions, if any, for ancillary and post-trial care, and for compensation to those who suffer harm from trial participation	10		
Dissemination policy	31a	Plans for investigators and sponsor to communicate trial results to participants, healthcare professionals, the public, and other relevant groups (eg, via publication, reporting in results databases, or other data sharing arrangements), including any publication restrictions	10		
	31b	Authorship eligibility guidelines and any intended use of professional writers	10-11		
	31c	Plans, if any, for granting public access to the full protocol, participant-level dataset, and statistical code	NA		
Appendices					
Informed consent materials	32	Model consent form and other related documentation given to participants and authorised surrogates	NA		
Biological specimens	33	Plans for collection, laboratory evaluation, and storage of biological specimens for genetic or molecular analysis in the current trial and for future use in ancillary studies, if applicable	NA		

[a] It is strongly recommended that this checklist be read in conjunction with the SPIRIT 2013 Explanation & Elaboration for important clarification on the items.
[b] Indicates page numbers to be completed by authors during protocol development.

Cite as:

For peer review only

BMJ Open

Determining the impact of an artificial intelligence tool on the management of pulmonary nodules detected incidentally on computed tomography (DOLCE) study protocol: a prospective, non-interventional multi-centre United Kingdom study

Journal:	*BMJ Open*
Manuscript ID	bmjopen-2023-077747.R2
Article Type:	Protocol
Date Submitted by the Author:	27-Nov-2023

Complete List of Authors:	O'Dowd, Emma; Nottingham University Hospitals NHS Trust<br>Berovic, Marko; King's College Hospital NHS Foundation Trust<br>CALLISTER, Matthew; Leeds Teaching Hospitals NHS Trust<br>Chalitsios, Christos ; University of Nottingham<br>Chopra, Disha; Optellum Ltd<br>Das, Indrajeet; University Hospitals of Leicester NHS Trust<br>Draper, Adrian; St George's Hospital, Respiratory Medicine<br>Garner, Justin; Royal Brompton and Harefield Hospitals<br>Gleeson, Fergus; Oxford University Hospitals NHS Foundation Trust<br>Janes, Sam; University College London<br>Kennedy, Martyn; Leeds Teaching Hospitals NHS Trust,<br>Lee, Richard; Royal Marsden Hospital NHS Trust<br>Mauri, Fabrizio; Optellum Ltd<br>McKeever, Tricia; University of Nottingham<br>McNulty, William; King's College Hospital NHS Foundation Trust<br>Murray, James; Royal Free London NHS Foundation Trust<br>Nair, Arjun; University College Hospital<br>Park, John; Oxford University Hospitals NHS Foundation Trust<br>Rawlinson, Janette; National Cancer Research Institute, Consumer forum, NCRI CSG (lung) subgroup, BTOG Steering Committee, NHSE CEG;<br>Sagoo, Gurdeep; University of Leeds, Academic Unit of Health Economics<br>Scarsbrook, Andrew; Leeds Teaching Hospitals NHS Trust<br>Shah, Pallav; Royal Brompton and Harefield NHS Foundation Trust<br>Sudhir, Rajini; University Hospitals of Leicester NHS Trust<br>Talwar, Ambika; Oxford University Hospitals NHS Foundation Trust<br>Thakrar, Ricky; University College London Hospitals NHS Foundation Trust<br>Watkins, Johnathan; Optellum Ltd<br>Baldwin, David; Nottingham University Hospitals NHS Trust

**Primary Subject Heading**:	Respiratory medicine
Secondary Subject Heading:	Diagnostics, Oncology, Radiology and imaging, Respiratory medicine
Keywords:	Clinical Trial, Chest imaging < RADIOLOGY & IMAGING, RADIOLOGY &

	IMAGING, Computed tomography < RADIOLOGY & IMAGING, Respiratory tract tumours < THORACIC MEDICINE

SCHOLARONE™ Manuscripts

Title

Determining the impact of an artificial intelligence tool on the management of pulmonary nodules detected incidentally on computed tomography (DOLCE) study protocol: a prospective, non-interventional multi-centre United Kingdom study.

Emma L O'Dowd[1], Marko Berovic[2], Matthew Callister[3], Christos V. Chalitsios[4], Disha Chopra[5], Indrajeet Das[6], Adrian Draper[7], Justin L Garner[8], Fergus Gleeson[9], Sam Janes[10], Martyn Kennedy[3], Richard Lee[11], Fabrizio Mauri[5], Tricia McKeever[4], William McNulty[2], James Murray[12], Arjun Nair[13], John Park[9], Janette Rawlinson[14], Gurdeep S Sagoo[15], Andrew Scarsbrook[3], Pallav L Shah[8], Rajini Sudhir[6], Ambika Talwar[9], Ricky Thakrar[13], Johnathan Watkins[5], David R Baldwin[1]

1. Nottingham University Hospitals NHS Trust, Nottingham, NG5 1PB

2. King's College Hospital NHS Foundation Trust, London SE5 9RS

3. Leeds Teaching Hospitals NHS Trust, Leeds LS9 7TF

4. University of Nottingham, Nottingham, NG5 1PB

5. Optellum Ltd, Oxford Centre For Innovation, New Road, Oxford, England, OX1 1BY

6. University Hospitals of Leicester NHS Trust, Leicester, Leicester, LE3 9QP

7. St George's University Hospitals NHS Foundation Trust, London, SW17 0QT

8. Royal Brompton and Harefield hospitals, London SW3 6NP

9. Oxford University Hospitals NHS Trust, Oxford, UK

10. Lungs for Living Research Centre, University College London, London, UK

11. The Royal Marsden NHS Foundation Trust, London SW3 6JJ

12. Royal Free London NHS Foundation Trust, London NW3 2QG

13. University College London Hospitals NHS Foundation Trust, London

14. Patient representative, National Cancer Research Institute (NCRI), Consumer forum, NCRI CSG (Lung) Subgroup, BTOG Steering Committee, NHSE CEG, UK

15. University of Newcastle, Newcastle upon Tyne

Corresponding author: Emma L O'Dowd (emma.o'dowd@nottingham.ac.uk)

For peer review only - http://bmjopen.bmj.com/site/about/guidelines.xhtml

Abstract

Introduction

In a small percentage of patients, pulmonary nodules found on computed tomography (CT) scans are early lung cancers. Lung cancer detected at an early stage has a much better prognosis. The British Thoracic Society (BTS) guideline on managing pulmonary nodules recommends using multivariable malignancy risk prediction models to assist in management. Whilst these guidelines seem to be effective in clinical practice, recent data suggest that artificial intelligence (AI)-based malignant-nodule prediction solutions might outperform existing models.

Methods and analysis

This study is a prospective, observational multicentre study to assess the clinical utility of an AI-assisted CT-based lung cancer prediction tool (LCP) for managing incidental solid and part solid pulmonary nodule patients versus standard care. Two thousand patients will be recruited from 12 different UK hospitals. The primary outcome is the difference between standard care and LCP-guided care in terms of the rate of benign-nodules and cancer patients discharged straight after the assessment of the baseline CT scan. Secondary outcomes investigate adherence to clinical guidelines, other measures of changes to clinical management, patient outcomes, and cost-effectiveness.

Ethics and dissemination

This study has been reviewed and given a favourable opinion by the South Central - Oxford C Research Ethics Committee in UK (REC reference number: 22/SC/0142).

Study results will be available publicly following peer-reviewed publication in open-access journals. A Patient and Public Involvement (PPI) group's workshop is planned before the study results will be available to discuss best methods to disseminate the results. Study results will also be fed back to participating organisations to inform training and procurement activities.

Trial registration number

The study is registered in Clinicaltrials.gov, number: NCT05389774

Strengths and limitations

- This is a prospective study examining the use of artificial intelligence (AI)-based computer-aided diagnosis (CADx) for pulmonary nodules
- This study will examine the clinical utility of an AI-based CADx tool for pulmonary nodule management
- The study is non-interventional in nature
- The analysis of some of the secondary objectives is exploratory due to potential sample size restrictions

Keywords

Clinical trial; chest imaging; radiology & imaging; computed tomography; respiratory tract tumours

For peer review only - http://bmjopen.bmj.com/site/about/guidelines.xhtml

Introduction

From 2017 to 2019, lung cancer caused 34,771 deaths in the UK per annum, more than prostate and bowel cancer combined (28,846), the next most common causes of cancer death. Over the same period, there were 24% more deaths in women from lung cancer than from breast malignancy (14,140 vs 11,415) [1]. This is because almost three-quarters of people with lung cancer present with stage III or IV disease when the prognosis is poor despite recent advances in systemic anti-cancer treatment. Lung cancer detected at an early stage, usually by thoracic computed tomography (CT), has a much better prognosis. Screening with low-dose CT thorax has recently been confirmed to reduce lung cancer and all-cause mortality [2–4]. Central to the success of screening is the accurate management of pulmonary nodules, which may represent early-stage lung cancer, but are far more often benign. Pulmonary nodules are also a common finding on CT scans done for other reasons. In some settings, this may yield more early lung cancers than screening. Identifying malignant pulmonary nodules on CT scans is the primary way to detect lung cancer at stage I, when the prognosis is best. By definition, isolated pulmonary nodules without other lung changes, are stage I if proven to be lung cancer. Currently, most nodules are detected outside screening programmes in the UK, providing an important rationale for this study to address the effective management of these incidentally detected nodules.

The British Thoracic Society (BTS) guideline on managing pulmonary nodules was published in 2015 [5]. This guidance was the first to recommend semi-automated volumetry as the standard for nodule measurement and the use of multivariable malignancy risk prediction models to assist in management. Whilst these guidelines seem to be effective in clinical practice [6], recent data suggest that artificial intelligence (AI)-based malignant-nodule prediction solutions appear to outperform existing multivariable models [7,8]. This could result in a considerable reduction in the number of follow-up CT, which is much needed in the NHS, where current and forecasted demand for radiology services exceeds capacity [9,10].

Optellum Ltd has developed and extensively validated an AI-based computer-aided diagnostic (CADx) tool for lung cancer prediction (LCP) in pulmonary nodules, which has UK Conformity Assessed marking under UK medical device regulations. The AI model underpinning the LCP is a convolutional neural network (CNN), referred to hereafter as the 'LCP-CNN'. LCP-CNN was derived and internally validated using data from diverse populations from the US and UK. The model was then externally validated in two independent cohorts of patients with incidentally detected pulmonary nodules, amounting to over 1,500 patients in aggregate [7,8]. The LCP-CNN demonstrated greater accuracy in these studies than the Brock and Mayo malignancy risk models (used in managing incidentally detected nodules in the UK and US, respectively). The product is provided within the "Virtual Nodule Clinic" (VNC), a software device used in the tracking, assessment and characterization of incidentally detected pulmonary nodules.

The study aims to assess this new AI tool in a prospective real-world setting in the UK. This will be done by calculating malignancy risk using LCP and measuring how management would differ if it were governed by the risk provided. The aim is to provide evidence as to whether LCP should be the new standard of care in pulmonary nodule management.

For peer review only - http://bmjopen.bmj.com/site/about/guidelines.xhtml

Methods and analysis

The study acronym is "DOLCE" (Determining the impact of Optellum's LCP artificial intelligence solution on service utilisation, health Economics and patient outcomes).

Design and setting

DOLCE is a multi-centre prospective observational cohort study recruiting patients with 5-30 mm solid and part-solid pulmonary nodules incidentally detected on thoracic CT scans performed as part of routine practice. The study aims to recruit 2,000 patients from 12 centres.

Patients will be asked to consent to and be enrolled at twelve acute NHS hospital trusts in England. A recruitment period of one year is planned, with one year of follow-up to collect data and ascertain the ground-truth diagnosis of recruited patients.

The start date was March 2023 with the planned recruitment end date being February 2024. With one year of follow up, this will mean a planned end date of February 2025.

Clinical pathway

This is a non-interventional study. There will be no anticipated change to the standard of care the recruited patients receive throughout the study's duration. The potential impact of AI-modified management will be assessed by comparing routine clinical management with that which would have been conditional upon the LCP risk score. The scores will be provided to clinicians after routine management decisions have been made, either during the same MDT meeting or at a separate research meeting. A hypothetical clinical decision based on the LCP score will then be recorded.

Study population

Patients who have undergone CT that has detected a pulmonary nodule and was performed for a reason other than suspicion of lung cancer will be reviewed for study eligibility (Table 1).

Table 1: Patient eligibility criteria

**Inclusion criteria**
Patients aged 35 years or over
Patients with a baseline CT study with at least one incidentally detected solid or part-solid pulmonary nodule that is 5-30mm in maximum axial diameter for the whole lesion, measured using manual electronic callipers, and that is not fully calcified. The solid component of the part-solid nodule must be ≥ 80% of the total nodule size.
Patients with a baseline CT study which includes at least one series with acquisition parameters which meet VNC requirements
The patient has a baseline CT study that includes at least one series that comprises at least one full-inspiration breath-hold scan without a high degree of contrast media and does not exhibit quality issues (e.g., motion artefacts)
**Exclusion criteria**
Patients who received a diagnosis of cancer in the last 5 years

For peer review only - http://bmjopen.bmj.com/site/about/guidelines.xhtml

Patients who have thoracic implants that adversely impact the imaging appearances of the nodule
Patients who have more than five reported pulmonary nodules of any size or type, excluding fully calcified nodules (a marker of prior granulomatous infection or metastatic lung cancer)
Patients who have one or more additional nodules that are already undergoing follow-up according to pulmonary nodule management standard care
Patients who have one or more additional nodules that are pure ground glass opacity (GGO) of 5mm in maximum axial diameter for the whole lesion measured using manual electronic callipers
Patients who have one or more additional nodules >30mm in maximum axial diameter for the whole lesion measured using manual electronic callipers.
Patients with part-solid nodules where the solid component is < 80% of the total nodule size.

AI model

The output of the LCP is a risk score that is an integer from 1 to 10 and forms part of Optellum's VNC patient management software. A score of 1 indicates the nodule is highly likely to be benign, while a score of 10 is most likely to be malignant. The AI model underpinning the LCP is the LCP-CNN, the raw score available to researchers as a continuous value from 0 to 100, as previously reported. LCP-CNN was derived and internally validated using data from diverse populations from the US and UK. The model was then externally validated in two independent cohorts of patients with incidentally detected pulmonary nodules, amounting to over 1,500 patients in aggregate [7,8].

In this study, the LCP will be compared to standard care in three ways:

1. LCP as an integer (1-10) risk score to aid physician decision-making (physician guided by AI score)
2. LCP-CNN alone as a threshold-based rule-out recommendation (AI binary decision to discharge or not)
3. LCP-CNN combined with a threshold-based rule-out recommendation to aid physician decision-making (physician guided by AI with binary recommendation to discharge or not)

Data collection

Study teams at the twelve NHS Trusts from which patients will be recruited will identify eligible patients and obtain informed consent. Consent will be obtained verbally (for patients who would not normally come on-site as part of standard care) or via written consent. Consent will be sought to analyse standard-care CT scans, associated clinical meta-data collection, and the health economics assessment.

Once a patient has given consent and enrolled into the study, their baseline CT scan will be sent to VNC. Authorised research staff will then log into VNC, select the nodules of interest, and record the LCP score for that nodule. The local standard nodule service decision-makers will provide a standard-care decision for eligible, consented patients via their usual nodule

For peer review only

For peer review only - http://bmjopen.bmj.com/site/about/guidelines.xhtml

management pathway. The standard-care decision for eligible consented patients will then be recorded in an electronic Case Report Form (eCRF) without knowing the LCP score.

To assess LCP under scenario #1 as described under *AI model*, the clinical team will thereafter be given the LCP score and relevant patient data in the nodule MDT or as part of a separate research meeting. At this point, the clinical team should remain blinded to the outcome of any actual-care investigation (e.g., PET/CT scan, biopsy etc.). Their decision on the next management step, had they been guided by the LCP score, will then be recorded in the eCRF. The clinical team will have the option to modify management at this point. Any modification of management will be recorded in the eCRF.

Subsequent follow-up images for each patient will be sent to the VNC over the course of their nodule follow-up, and the final nodule diagnosis will be recorded. Since standard patient care can sometimes deviate from BTS guidelines, all participating sites will also collect information on patient and nodule characteristics necessary to follow the BTS guidelines. In this way, AI-guided management can be compared with BTS-compliant management. Other data points necessary to evaluate service utilisation (e.g. additional scans, biopsies, and treatments for lung nodules or lung cancers) will also be recorded in the eCRF.

In line with SPIRIT-AI guidelines, performance errors that would have happened if the LCP or LCP-guided decision making were part of standard care will be analysed and reported as outcome measures for the primary objective of this study.

Evaluation of LCP as threshold-based tests (scenarios #2 and #3 as described under *AI model*), which require access to LCP-CNN values, will be performed as a batch at the end of the study follow-up period. The threshold will be defined prospectively based on values from training datasets of similar patient populations. The use of the recommendation with physicians making the decision will be evaluated via a separate research meeting at the end of the 12-month follow-up period.

For cost-effectiveness modelling, quality of life data will be collected by inviting patients to complete an EQ-5D-5L quality of life questionnaire at two-time points: a) as close as possible to the baseline CT scan on which their pulmonary nodule was detected; and b) at a 1-year timepoint afterwards. A participant diary about primary care usage will also be collected at the one-year mark.

Ground truth diagnosis

Patients will be classified into whether they have benign or malignant nodules as follows:

1. Benign-nodule patients:
    a. By resolution or stability of all qualifying incidental pulmonary nodules as determined from volumetry-based measurement on imaging follow-up over 12 months
    b. By histopathology from biopsy or surgery of all qualifying incidental pulmonary nodules (within 12 months of baseline CT scan)
2. Malignant-nodule patients:
    a. By histopathology from biopsy or surgery (within 12 months of baseline CT scan) of at least one qualifying incidental pulmonary nodule
3. Probable benign-nodule patients:

For peer review only - http://bmjopen.bmj.com/site/about/guidelines.xhtml

a. By resolution, stability, or shrinkage of qualifying incidental pulmonary nodules that are not in ground-truth outcomes #1 or #2 above as determined from volumetry-based measurement on imaging follow-up conducted between 3-12 months after the baseline CT scan
b. By resolution, stability, or shrinkage of qualifying incidental pulmonary nodules that are not in ground-truth outcomes 1 or 2 above as determined from diameter-based measurement on imaging follow-up conducted 12 months after the baseline CT scan (supplemented with a volume-based measurement of the nodule that will be performed for research purposes)

4. Probable malignant-nodule patients:
   a. By clinical diagnosis of lung cancer, in whom a biopsy or resection is not possible, based on high clinical probability and who are registered at a lung cancer MDT/and/or receive non-surgical treatment (SABR/ radiotherapy/ microwave ablation)

Volumetry-based measurement of nodule stability – and hence a benign diagnosis – will be satisfied if the lung nodule volume on the follow-up CT scan was <125% of the lung nodule volume on the baseline CT scan.

If the standard care at a particular site does not incorporate volumetry-based measurements, the volumetric measurement will be applied as part of the research study to the relevant follow-up CT scans.

Any lung nodule patients who do not fall into either of the two ground-truth outcomes 1 or 2 above will be considered to have an indeterminate diagnosis at the time of analysis and will be analysed with and without the inclusion of the ground-truth outcomes in 3 and 4.

Outcome measures

The outcome measures for the primary and secondary objectives are outlined in Table 2.

Table 2: Study objectives and outcome measures

Objective	Outcome measure
**Primary**:<br>Determine the potential effect of the LCP on discharge	The measured difference between standard care and the three LCP-based options described under *AI model* for:<br>• Number and percentage of cancer patients discharged (straight after assessment of the baseline scan)<br>• Number and percentage of benign-nodule patients discharged (straight after assessment of the baseline scan)
**Secondary**:<br>Determine the potential effect of the LCP on possible	The measured difference between standard care and the three LCP-based options described under *AI model*:<br>• Number and percentage of patients for whom a validated risk model (Brock or LCP) is used to guide the next clinical management step (i.e., counting the instances where Brock is not used or where LCP is not possible to compute, or it is ignored)

For peer review only - http://bmjopen.bmj.com/site/about/guidelines.xhtml

adherence to clinical guidelines	
**Secondary**:<br>Determine the potential effect of the LCP on overall clinical management, as well as scan and procedure utilisation	Number and percentage of cancer patients for whom there would have been a change in clinical management according to each of the three LCP-based options described under *AI model* compared with actual care (correctly for more aggressive management and incorrectly for less aggressive management).<br>Number and percentage of benign-nodule patients for whom there would have been a change in clinical management according to each of the three LCP-based options described under *AI model* compared with actual care (incorrectly by more aggressive management and correctly for less aggressive management).<br>The measured difference between standard care and the three LCP-based options described under *AI model* for:<br>• Number and percentage of CT scans and PET/CT scans performed on benign-nodule patients<br>• Number and percentage of non-surgical biopsies performed on benign-nodule patients<br>• Number and percentage of surgical excisions on benign-nodule patients
**Secondary**:<br>Determine the potential effect of LCP-guided care versus standard care on patient outcomes	The measured difference between standard care and the three LCP-based options described under *AI model* for:<br>• Number and percentage of thoracic, respiratory, or vascular events related to biopsies or surgical excisions for lung nodules or suspected lung cancer occurring within 30 days of the procedure on benign-nodule patients<br>• Number and percentage of lung cancers stratified by stage<br>• Time in days between nodule detection and lung cancer diagnosis
**Secondary**:<br>Determine the potential health-economic effect of LCP-guided care versus standard care	The measured difference between standard care and the three LCP-based options described under *AI model* for:<br>• The composite standardised GBP costs of all healthcare-related activity for lung nodules or suspected lung cancer<br>• Health-Related Quality of Life (using EQ5D5L to generate QALYs)<br>• Estimate cost-effectiveness (Incremental costs per QALY gained)

Statistical analysis

Sample size

For calculations relating to the primary objectives of a measured difference between standard care and LCP for*:*

For peer review only

For peer review only - http://bmjopen.bmj.com/site/about/guidelines.xhtml

- Number and percentage of cancer patients discharged: results from thresholds defined for discharge in a previous study are used [7]. Based on previous data within the cancer group, assuming for the discordant pairs that the Brock model threshold (representing the standard of care) identified 0.4% of cancers for which the LCP threshold would have recommended discharge, and the LCP threshold correctly identified 2.6% of cancers for which the Brock model threshold would have recommended discharge, then using McNemar's test with 90% power and a significance level of 0.05, 659 participants would be needed. However, with a sample size of 2000, there is 90% power to detect a difference between the methods if the percentages were as small as 1.0% and 2.3%, respectively.
- Number and percentage of benign-nodule patients discharged: again, results from thresholds defined for discharge in a previous study are used [7]. Based on previous data within the benign group, assuming for the discordant pairs that 17.0% of benign nodules are recommended for discharge by the LCP threshold but not by the Brock model threshold. In comparison, 13.5% are recommended for discharge by the Brock model threshold but not by the LCP threshold; there is 80% power to detect a significant difference between the two tests with 2,026 individuals using McNemar's test. This equates to an odds ratio of 1.26

Sample size calculations were performed using R statistical software (version 4.1.2; R Foundation for Statistical Computing, Vienna, Austria).

Statistical analysis

Statisticians will carry out all analyses. For each outcome measure, the following will be reported:

- The number of participants included in the analysis, stratified by the relevant group
- Mean or median (SD or IQR) for continuous outcomes, and numbers and proportions (percentage) for binary outcomes
- An effect size (odds ratio) with a 95% confidence interval
- A two-sided p-value

For both primary objectives, measured differences between standard care and the LCP options for cancer patients diagnosed and benign-nodule patients discharged will be analysed using conditional logistic regression. Statistical significance will be assessed with a Wald test. Secondary outcomes will be described and analysed according to their distribution. All outcomes will have an exact p-value provided for them so the reader can access the data for significance level, so correction for multiple testing should not be needed.

Health economics analysis

The economic analysis will estimate the expected costs and expected QALYs gained from standard care, LCP, and LCP-guided care over patient lifetime from the perspective of the UK NHS. Incremental analyses will identify the most cost-effective permutation with exploratory analyses undertaken to determine the uncertainty around measures of cost-effectiveness. Costs will be calculated as quantities of resource use multiple by appropriate unit costs (e.g. NHS Reference Costs) and will be specific to a year to be determined at time of analysis. A HEAP (health economics analysis plan) will be produced prior to undertaking the analysis at the end of the study patient follow-up period.

For peer review only - http://bmjopen.bmj.com/site/about/guidelines.xhtml

Patient and public involvement

An extensive programme of patient and public involvement (PPI) input was arranged to provide input into the study objectives, design, materials and governance and planned to disseminate study results.

Specifically, a PPI representative, who sits on the Trial Management Group, provided input from study conception to developing study materials. This guidance and support will continue during the management and conduct of the study. An independent PPI contributor is a member of the trial steering committee (TSC). Further input was gathered from a group of PPI contributors selected from across the country with varying experiences of lung nodules, cancer or none from a patient and/or carer perspective during facilitated virtual workshops.

Input received to date has ranged from considerations of healthcare inequalities and other biases, awareness of AI generally and in healthcare, methods of and materials for participant recruitment and communication, and the design of any data collected during the study.

Ethics and dissemination

This study has been reviewed and given a favourable opinion by the South Central - Oxford C Research Ethics Committee in UK (REC reference number: 22/SC/0142).

Given the study's observational nature, we do not envisage any elevation to the harms or the risk of harm for patients. The sponsor and Chief Investigator have conducted a full risk assessment.

Patients will be asked to give their consent to participate in the study (including the analysis of standard-care CT scans, associated data collection and the healthcare economics assessment) by telephone or face-to-face when attending routine clinic appointments. No additional patient visits, scans or procedures are planned for the study. Patients not wishing to participate in the health economics assessment may still participate in the study's CT imaging analysis.

The findings of this study will be shared with the funder (NHS Accelerated Access Collaborative). Results will be presented at academic and clinical conferences. The study will result in at least one peer-reviewed publication of the findings in an international medical journal.

References

1 Cancer Research UK. Cancer mortality for common cancers. 2022.https://www.cancerresearchuk.org/health-professional/cancer-statistics/mortality/common-cancers-compared

2 Lindholt JS, Søgaard R. Lung-Cancer Screening and the NELSON Trial. *N Engl J Med* 2020;**382**:2164. doi:10.1056/NEJMc2004224

3 de Koning HJ, van der Aalst CM, de Jong PA, *et al.* Reduced Lung-Cancer Mortality with Volume CT Screening in a Randomized Trial. *N Engl J Med* 2020;**382**:503–13. doi:10.1056/NEJMoa1911793

For peer review only - http://bmjopen.bmj.com/site/about/guidelines.xhtml

4 National Lung Screening Trial Research Team, Aberle DR, Adams AM, *et al.* Reduced lung-cancer mortality with low-dose computed tomographic screening. *N Engl J Med* 2011;**365**:395–409. doi:10.1056/NEJMoa1102873

5 Callister MEJ, Baldwin DR, Akram AR, *et al.* British Thoracic Society guidelines for the investigation and management of pulmonary nodules. *Thorax* 2015;**70 Suppl 2**:ii1–54. doi:10.1136/thoraxjnl-2015-207168

6 Balata H, Ruparel M, O'Dowd E, *et al.* Analysis of the baseline performance of five UK lung cancer screening programmes. *Lung Cancer* 2021;**161**:136–40. doi:10.1016/j.lungcan.2021.09.012

7 Baldwin DR, Gustafson J, Pickup L, *et al.* External validation of a convolutional neural network artificial intelligence tool to predict malignancy in pulmonary nodules. *Thorax* 2020;**75**:306–12. doi:10.1136/thoraxjnl-2019-214104

8 Massion PP, Antic S, Ather S, *et al.* Assessing the Accuracy of a Deep Learning Method to Risk Stratify Indeterminate Pulmonary Nodules. *Am J Respir Crit Care Med* 2020;**202**:241–9. doi:10.1164/rccm.201903-0505OC

9 The Royal College of Radiologists. RCR Clinical radiology census report 2021. 2021.

10 Maskell G. Why does demand for medical imaging keep rising? *BMJ* 2022;:o2614. doi:10.1136/bmj.o2614

Authors' contributions

All authors (EOD, DRB, JW, FM, MB, MC, CC, DC, ID, AD, JG, FG, SJ, MK, RL, TM, WM, JM, AN, JP, JR, GS, AS, PS, RS, AT, RT) contributed to the development and set up of the study. EOD, DRB, JW, FM wrote the manuscript, all authors (EOD, DRB, JW, FM, MB, MC, CC, DC, ID, AD, JG, FG, SJ, MK, RL, TM, WM, JM, AN, JP, JR, GS, AS, PS, RS, AT, RT) contributed to, reviewed and approved the final version of the manuscript.

Funding statement

This work is supported NHS Accelerated Access Collaborative. Grant number AI_AWARD02266. Sponsor organisation is Nottingham University Hospitals NHS Trust. The sponsor organisation and funder have had no role in study design.

Competing interests

FG reports grant funding from NIHR and Innovate UK, consultancy fees from Polarean Ltd and has shares in Optellum Ltd; SJ has received grant funding from GRAIL Ltd, consultancy fees/ honoraria from AstraZeneca and Chiesi, stock options in Optellum Ltd and his spouse is employed by AstraZeneca; RL is funded by the Royal Marsden NIHR BRC, Royal Marsden Cancer charity and SBRI (including QURE.AI). RL's institution receives compensation for time spent in a secondment role for the lung health check program and as a National Specialty Lead for the National Institute of Health and Care Research. He has received research funding from CRUK, Innovate UK (co-funded by GE Healthcare, Roche and Optellum), SBRI, RM Partners Cancer Alliance and NIHR (co-applicant in grants with Optellum). He has received

For peer review only - http://bmjopen.bmj.com/site/about/guidelines.xhtml

honoraria from CRUK; WM has received honoraria from Amgen; AN declares grants from Department of Health's NIHR Biomedical Research Centre's funding scheme and GRAIL, consulting fees from Aidence BV, Faculty Science LTD and MSD; Support for attending meetings from Takeda Limited; Advisory Board participation for Aidence BV and Faculty Science Ltd; JR has received support for travel and accommodation from BTOG, ERS, CRUK, EORTC, ESMO and NCRI; RT has received honoraria from Olympus Medical; DRB has grant from NHS Digital and had received honoraria from MSD and AstraZeneca; DC, FM and JW are Optellum Ltd employees and have share options in Optellum Ltd. The remaining authors declare no conflicts of interests.

Word count: 2700

For peer review only

For peer review only - http://bmjopen.bmj.com/site/about/guidelines.xhtml

SPIRIT-AI Checklist: Recommended items to address in a protocol and related documents for clinical trials evaluating AI interventions

Section		SPIRIT 2013 Item[a]	SPIRIT-AI Item		Addressed on Page No [b]
		Administrative Information			
Title	1	Descriptive title identifying the study design, population, interventions, and, if applicable, trial acronym	SPIRIT-AI 1(i) Elaboration	Indicate that the intervention involves artificial intelligence / machine learning and specify the type of model.	1
			SPIRIT-AI 1(ii) Elaboration	Specify the intended use of the AI intervention.	3
Trial registration	2a	Trial identifier and registry name. If not yet registered, name of intended registry	2		
	2b	All items from the World Health Organization Trial Registration Data Set	2		
Protocol version	3	Date and version identifier	NA		
Funding	4	Sources and types of financial, material, and other support	11		
Roles and responsibilities	5a	Names, affiliations, and roles of protocol contributors	1		
	5b	Name and contact information for the trial sponsor	11		
	5c	Role of study sponsor and funders, if any, in study design; collection, management, analysis, and interpretation of data; writing of the report; and the decision to submit the report for publication, including whether they will have ultimate authority over any of these activities	11		
	5d	Composition, roles, and responsibilities of the coordinating centre, steering committee, endpoint adjudication committee, data management team, and other individuals or groups overseeing the trial, if applicable (see Item 21a for data monitoring committee)	11		
		Introduction			
Background and rationale	6a	Description of research question and justification for undertaking the trial, including summary of relevant studies (published and unpublished) examining benefits and harms for each intervention	SPIRIT-AI 6a (i) Extension	Explain the intended use of the AI intervention in the context of the clinical pathway, including its purpose and its intended users (e.g. healthcare professionals, patients, public).	3
			SPIRIT-AI 6a (ii) Extension	Describe any pre-existing evidence for the AI intervention.	3

For peer review only

Cite as:

For peer review only - http://bmjopen.bmj.com/site/about/guidelines.xhtml

	6b	Explanation for choice of comparators	3			
Objectives	7	Specific objectives or hypotheses	3			
Trial design	8	Description of trial design including type of trial (eg, parallel group, crossover, factorial, single group), allocation ratio, and framework (eg, superiority, equivalence, noninferiority, exploratory)	3			
Methods: Participants, Interventions and Outcomes						
Study setting	9	Description of study settings (eg, community clinic, academic hospital) and list of countries where data will be collected. Reference to where list of study sites can be obtained		SPIRIT-AI 9 Extension	Describe the onsite and offsite requirements needed to integrate the AI intervention into the trial setting.	5, 6
Eligibility criteria	10	Inclusion and exclusion criteria for participants. If applicable, eligibility criteria for study centres and individuals who will perform the interventions (eg, surgeons, psychotherapists)		SPIRIT-AI 10 (i) Elaboration	State the inclusion and exclusion criteria at the level of participants.	4, 5
				SPIRIT-AI 10 (ii) Extension	State the inclusion and exclusion criteria at the level of the input data.	4, 5
Interventions	11a	Interventions for each group with sufficient detail to allow replication, including how and when they will be administered		SPIRIT-AI 11a (i) Extension	State which version of the AI algorithm will be used.	5
				SPIRIT-AI 11a (ii) Extension	Specify the procedure for acquiring and selecting the input data for the AI intervention.	5
				SPIRIT-AI 11a (iii) Extension	Specify the procedure for assessing and handling poor quality or unavailable input data.	4-7
				SPIRIT-AI 11a (iv) Extension	Specify whether there is human-AI interaction in the handling of the input data, and what level of expertise is required for users.	4-7
				SPIRIT-AI 11a (v) Extension	Specify the output of the AI intervention.	4-7
				SPIRIT-AI 11a (vi) Extension	Explain the procedure for how the AI intervention's output will contribute to decision-making or other elements of clinical practice.	4-7
	11b	Criteria for discontinuing or modifying allocated interventions for a given trial participant (eg, drug dose change in response to harms, participant request, or improving/worsening disease)	4-8			
	11c	Strategies to improve adherence to intervention protocols, and any procedures for monitoring adherence (eg, drug tablet return, laboratory tests)	4-8			
	11d	Relevant concomitant care and interventions that are permitted or prohibited during the trial	4-8			
Outcomes	12	Primary, secondary, and other outcomes, including the specific measurement variable (eg, systolic blood pressure), analysis metric (eg, change from baseline, final value, time to event), method of aggregation (eg, median, proportion), and time point for each outcome. Explanation of the clinical relevance of chosen efficacy and harm outcomes is strongly recommended	7/8			

Cite as:

For peer review only

For peer review only - http://bmjopen.bmj.com/site/about/guidelines.xhtml

Participant timeline	13	Time schedule of enrolment, interventions (including any run-ins and washouts), assessments, and visits for participants. A schematic diagram is highly recommended (see Figure)	4-8		
Sample size	14	Estimated number of participants needed to achieve study objectives and how it was determined, including clinical and statistical assumptions supporting any sample size calculations	8-9		
Recruitment	15	Strategies for achieving adequate participant enrolment to reach target sample size	4-9		
Methods: Assignment of Interventions (For Controlled Trials)					
Sequence generation	16A	Method of generating the allocation sequence (eg, computer-generated random numbers), and list of any factors for stratification. To reduce predictability of a random sequence, details of any planned restriction (eg, blocking) should be provided in a separate document that is unavailable to those who enrol participants or assign interventions	NA		
Allocation concealment mechanism	16b	Mechanism of implementing the allocation sequence (eg, central telephone; sequentially numbered, opaque, sealed envelopes), describing any steps to conceal the sequence until interventions are assigned	NA		
Implementation	16c	Who will generate the allocation sequence, who will enrol participants, and who will assign participants to interventions	NA		
Blinding (masking)	17a	Who will be blinded after assignment to interventions (eg, trial participants, care providers, outcome assessors, data analysts), and how	NA		
	17b	If blinded, circumstances under which unblinding is permissible, and procedure for revealing a participant's allocated intervention during the trial	NA		
Methods: Data Collection, Management, And Analysis					
Data collection methods	18a	Plans for assessment and collection of outcome, baseline, and other trial data, including any related processes to promote data quality (eg, duplicate measurements, training of assessors) and a description of study instruments (eg, questionnaires, laboratory tests) along with their reliability and validity, if known. Reference to where data collection forms can be found, if not in the protocol	4-9		
	18b	Plans to promote participant retention and complete follow-up, including list of any outcome data to be collected for participants who discontinue or deviate from intervention protocols	4-9		
Data management	19	Plans for data entry, coding, security, and storage, including any related processes to promote data quality (eg, double data entry; range checks for data values). Reference to where details of data management procedures can be found, if not in the protocol	4-9		

Cite as:

For peer review only - http://bmjopen.bmj.com/site/about/guidelines.xhtml

SPIRIT-AI

Statistical methods	20a	Statistical methods for analysing primary and secondary outcomes. Reference to where other details of the statistical analysis plan can be found, if not in the protocol	8-9		
	20b	Methods for any additional analyses (eg, subgroup and adjusted analyses)	8-9		
	20c	Definition of analysis population relating to protocol non-adherence (eg, as randomised analysis), and any statistical methods to handle missing data (eg, multiple imputation)	8-9		
Methods: Monitoring					
Data monitoring	21a	Composition of data monitoring committee (DMC); summary of its role and reporting structure; statement of whether it is independent from the sponsor and competing interests; and reference to where further details about its charter can be found, if not in the protocol. Alternatively, an explanation of why a DMC is not needed	9-10		
	21b	Description of any interim analyses and stopping guidelines, including who will have access to these interim results and make the final decision to terminate the trial	10		
Harms	22	Plans for collecting, assessing, reporting, and managing solicited and spontaneously reported adverse events and other unintended effects of trial interventions or trial conduct	SPIRIT-AI 22 Extension	Specify any plans to identify and analyse performance errors. If there are no plans for this, explain why not.	5-10
Auditing	23	Frequency and procedures for auditing trial conduct, if any, and whether the process will be independent from investigators and the sponsor	10		
Ethics and Dissemination					
Research ethics approval	24	Plans for seeking research ethics committee/institutional review board (REC/IRB) approval	10		
Protocol amendments	25	Plans for communicating important protocol modifications (eg, changes to eligibility criteria, outcomes, analyses) to relevant parties (eg, investigators, REC/IRBs, trial participants, trial registries, journals, regulators)	9-10		
Consent or ascent	26a	Who will obtain informed consent or assent from potential trial participants or authorised surrogates, and how (see Item 32)	4-9		
	26b	Additional consent provisions for collection and use of participant data and biological specimens in ancillary studies, if applicable	NA		
Confidentiality	27	How personal information about potential and enrolled participants will be collected, shared, and maintained in order to protect confidentiality before, during, and after the trial	4-9		
Declaration of interests	28	Financial and other competing interests for principal investigators for the overall trial and each study site	11-12		

Cite as:

For peer review only - http://bmjopen.bmj.com/site/about/guidelines.xhtml

SPIRIT-AI

Access to data	29	Statement of who will have access to the final trial dataset, and disclosure of contractual agreements that limit such access for investigators	SPIRIT-AI 29 Extension	State whether and how the AI intervention and/or its code can be accessed, including any restrictions to access or re-use.	4-10
Ancillary and post-trial care	30	Provisions, if any, for ancillary and post-trial care, and for compensation to those who suffer harm from trial participation	10		
Dissemination policy	31a	Plans for investigators and sponsor to communicate trial results to participants, healthcare professionals, the public, and other relevant groups (eg, via publication, reporting in results databases, or other data sharing arrangements), including any publication restrictions	10		
	31b	Authorship eligibility guidelines and any intended use of professional writers	10-11		
	31c	Plans, if any, for granting public access to the full protocol, participant-level dataset, and statistical code	NA		
Appendices					
Informed consent materials	32	Model consent form and other related documentation given to participants and authorised surrogates	NA		
Biological specimens	33	Plans for collection, laboratory evaluation, and storage of biological specimens for genetic or molecular analysis in the current trial and for future use in ancillary studies, if applicable	NA		

[a] It is strongly recommended that this checklist be read in conjunction with the SPIRIT 2013 Explanation & Elaboration for important clarification on the items.
[b] Indicates page numbers to be completed by authors during protocol development.

Cite as:

For peer review only
